# Tailoring Alginate Hydrogels via Precoordinated Lanthanide Complexes as Dynamic Cross-Linkers

**DOI:** 10.1021/acs.biomac.5c02784

**Published:** 2026-05-05

**Authors:** Yu-Chia Su, Li-Hsin Chang, Tai-Lin Wu, Po-Heng Lin, Yi-Cheun Yeh

**Affiliations:** † Institute of Polymer Science and Engineering, 33561 National Taiwan University, Taipei 10617, Taiwan; ‡ Department of Chemistry, 34916 National Chung Hsing University, 250 Kuo Kuang Rd., Taichung 402, Taiwan

## Abstract

Lanthanides are attractive ionic cross-linkers for connecting hydrophilic polymers to form luminescent hydrogels. However, lanthanide-complexed networks often lack mechanical robustness. Here, precoordinated lanthanide complexes are introduced as both luminophores and dynamic cross-linkers to fabricate mechanically stable alginate hydrogels. Three types of precoordinated lanthanide complexes featuring lanthanides of varying ionic sizes (i.e., terbium (Tb^3+^), europium (Eu^3+^), and samarium (Sm^3+^)) spontaneously cross-link alginate through electrostatic interactions to form Alg-Ln hydrogels. The Alg-Sm hydrogel exhibits a shorter gelation time, a denser network, and superior mechanical strength compared to Alg-Eu and Alg-Tb hydrogels. These results are attributed to the larger volume of the Sm complex, which increases the spacing between polymer chains and promotes the formation of more electrostatic cross-linking points. Additionally, the Alg-Eu hydrogel serves as a promising luminescent sensor for copper ions. Taken together, the use of precoordinated lanthanide complexes as cross-linking agents enables tailored customization of hydrogel structures and properties.

## Graphical abstract

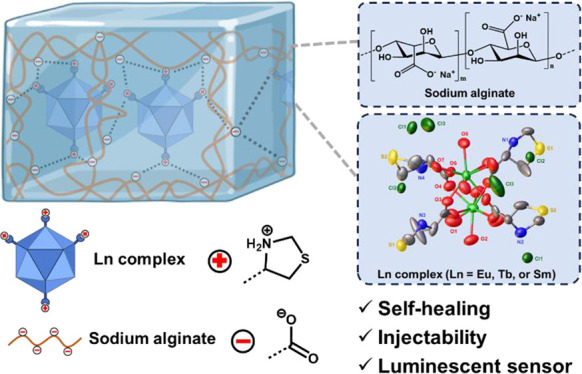

## 1 Introduction

Alginate hydrogels have attracted considerable attention due to their biocompatibility, nontoxicity, and ease of gelation under mild conditions, making them suitable for applications such as drug delivery,^1–3^ sensing,^4–6^ tissue engineering,^7,8^ and wound healing.^9,10^ In general, alginate hydrogels can be prepared through ionic cross-linking,^11–14^ covalent bonding,^15^ physical entanglement,^16^ phase transition,^17^ cell cross-linking,^18^ and free radical polymerization.^1^ Among these methods, ionic cross-linking with divalent or polyvalent cations is most commonly used due to its simplicity and tunability in the hydrogel properties. For example, Song et al. reported the preparation of sodium alginate/krill protein/polyacrylamide hydrogel (SA/AKP/PAM) through ionic cross-linking using multivalent cations (i.e., ferric (Fe^3+^), calcium (Ca^2+^), strontium (Sr^2+^), zinc (Zn^2+^), and barium (Ba^2+^)) in agar molds to regulate the gelation process as well as generate uniform network structures.^19^ In addition, Liu et al. developed a biomimetic alginate hydrogel modified with chondroitin sulfate for the construction of standardized tumor metastasis models and cancer drug screening.^20^ In this system, Ca^2+^ ion coordination facilitates cross-linking between alginate and chondroitin sulfate molecules, resulting in both conventional egg-box structures and novel asymmetric egg-box-like structures.

Recent advances in hydrogel technology have focused on incorporating lanthanide ions or their complexes as cross-linking agents for alginate to fine-tune the structures and properties of alginate hydrogels as well as generate luminescent alginate hydrogels. Lanthanide ions, known for their high coordination numbers and flexible bonding geometries, form stable complexes with the carboxylate groups of alginate, resulting in robust hydrogels with unique physicochemical properties. For example, Zhang et al. developed alginate hydrogels that contain covalently bound yttrium orthovanadate-europium (YVO_4_–Eu^3+^) for the detection of acetone.^21^ They also synthesized a terbium-based alginate hydrogel and incorporated tetrakis­(4-carboxyphenyl)­porphyrin (TCPP) as an antenna ligand to enhance luminescence.^22^ These novel hybrid materials have the potential to act as luminescent sensors for detecting Fe^3+^ with relative selectivity and high sensitivity. Ma et al. created two innovative organic–inorganic hybrid hydrogels through the self-assembly of alginate and lanthanide elements (i.e., europium (Eu^3+^) and terbium (Tb^3+^)), where luminescence can be turned on and off by the anthrax biomarker sodium dipicolinate.^23^ Kang et al. developed a self-assembly strategy to prepare luminescent hydrogels based on alginate and Tb^3+^ ions, using 5-sulfosalicylic acid (SSA) as a cofactor ligand to enhance the luminescence efficiency of the hydrogels and prolong the lifetime of luminescence.^24^ Recently, our group reported a novel luminescent triple-cross-linked hydrogel that incorporated Eu^3+^ ions into a double-cross-linked polymeric network of gelatin and alginate using the cotreatment of freeze-drying-swelling (FDS) and freeze–thawing (FT).^25^ The resulting hydrogels exhibited significant luminescent properties and mechanical strength, as well as presenting their potential applications in bacterial growth monitoring and copper ion detection.

Incorporation of lanthanide complexes into hydrogels at the molecular level has proven to be feasible and enables the production of luminescent hydrogels with well-defined properties. However, the inherently high-water content of hydrogels often quenches the luminescence of the lanthanide-complexed hydrogel network due to interactions with water molecules, where the O–H overtones of these water molecules act as efficient nonradiative relaxation pathways to deactivate the excited lanthanide ion.^26,27^ To address this limitation, the integration of precoordinated lanthanide complexes into hydrogels has emerged as a promising strategy for the development of highly luminescent hydrogels. This approach effectively minimizes the interference from water molecules and preserves the luminescent properties of the Ln-complexed network. Despite their potential, only a few reports have explored precoordinated complexes as cross-linkers for hydrogel formation. For example, Li et al. presented a simple approach to prepare lanthanide-based luminescent hydrogels by copolymerization of precoordinated lanthanide complexes (i.e., [Ln­(4-VDPA)_3_]^3–^ (Ln = Eu or Tb; VDPA= 4-vinylpyridine-2,6-dicarboxylic acid)), acrylamide (AM), and [2-(methacryloyloxy)­ethyl]­trimethylammonium chloride (DMC).^28^ The lanthanide complexes were connected to the polymeric network through both covalent bonding and electrostatic interactions, allowing the hydrogel to possess improved mechanical properties, thermal stability, and adhesion. In our previous study, aldehyde-terminated precoordinated lanthanide complexes (i.e., Ln­(PA)_3_ (Ln = Eu or Tb; PA= protocatechuic aldehyde)) were used as cross-linking agents to effectively connect polyethylenimine-modified gelatin (PG) via imine bonds.^29^ The Eu­(PA)_3_/PG hydrogel exhibited a denser internal structure and significantly stronger mechanical properties than the Tb­(PA)_3_/PG hydrogel due to the larger molecular size and higher cross-linking efficiency of the Eu­(PA)_3_ complex to PG. Nevertheless, the covalently cross-linked PAM/DMC-Ln­(DPA)_3_ network restricts its dynamic properties (e.g., shear-thinning and self-healing), and the limited luminescence of the precoordinated Ln­(PA)_3_ complexes also constrains the potential applications of the PG/Ln­(PA)_3_ hydrogels.

Here, we aim to advance the use of precoordinated lanthanide complexes for the formation of luminescent and mechanically stable alginate hydrogels by employing precoordinated lanthanide complexes as both luminophores and dynamic cross-linkers. Using tailorable lanthanide complexes of varying ionic sizes as dynamic cross-linkers for alginate can lead to hydrogels with definable structures and properties for the specific requirements of modern applications. In particular, the incorporation of lanthanide complexes in hydrogels via dynamic bonding mechanisms can significantly simplify the fabrication process through the fast and spontaneous cross-linking dynamics, as well as bring the dynamic features to the hydrogel network.

Compared to the reported alginate hydrogel studies, a series of lanthanide-containing alginate hydrogels were synthesized by cross-linking alginate (Alg) with amine-functionalized lanthanide (Ln) complexes through dynamic electrostatic interactions, forming Alg-Ln hydrogels (Scheme 1). The microstructures and properties of Alg-Ln hydrogels were systematically investigated by varying the Ln ions (i.e., Tb^3+^, Eu^3+^, and samarium (Sm^3+^)) in the lanthanide complexes, providing a promising strategy to fine-tune the networks and characteristics of luminescent alginate hydrogels. Alg-Eu lyophilized hydrogels also demonstrated the ability for metal ion sensing.

**Scheme 1:**
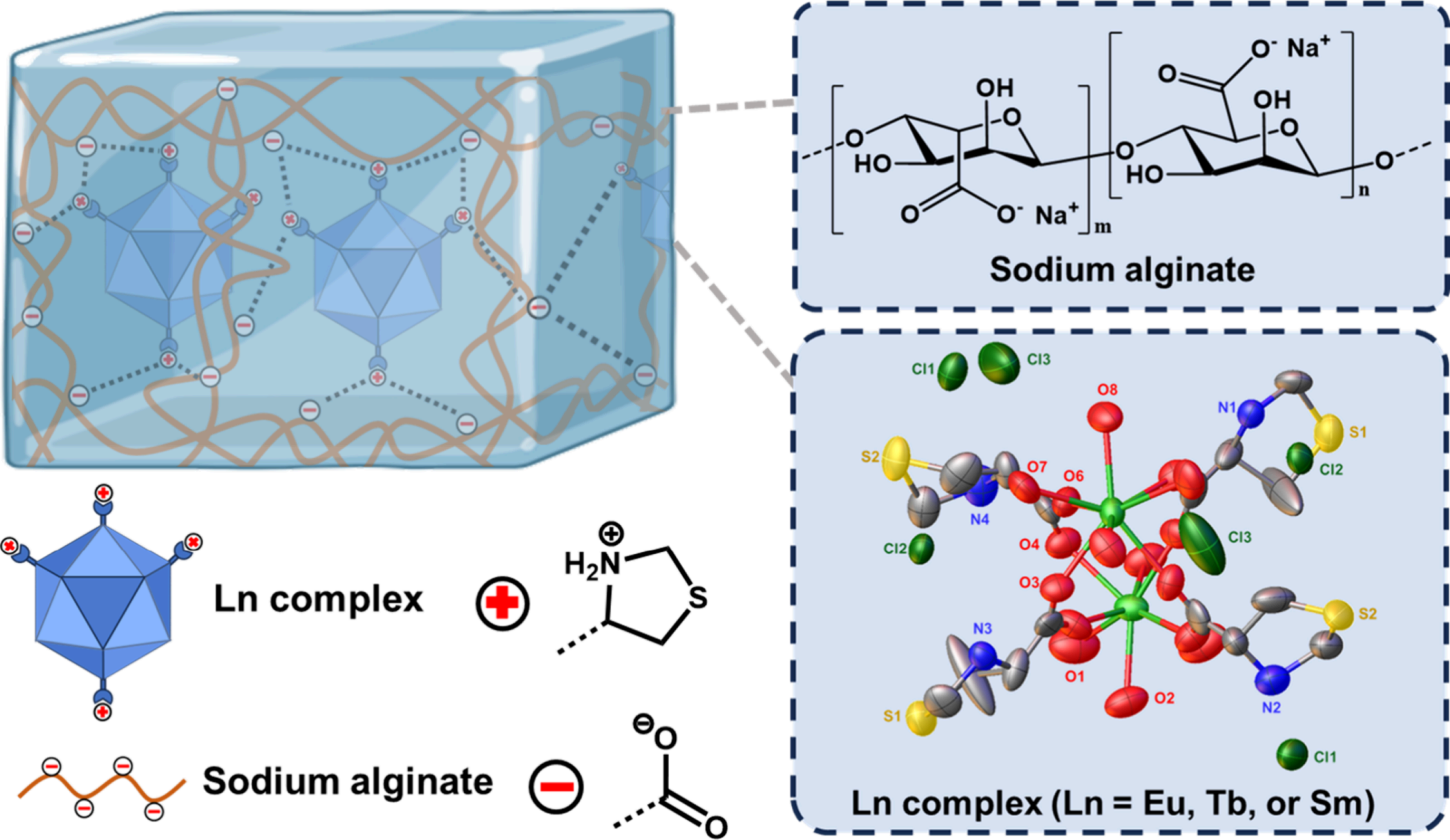
Schematic Illustration of the Composition and Internal Chemistry of the Alg-Ln Hydrogel

## 2 Materials and Methods

### 2.1 Materials

Europium­(III) chloride hexahydrate (EuCl_3_·6H_2_O, 99%), terbium­(III) chloride hexahydrate (TbCl_3_·6H_2_O, 99.9%), and samarium­(III) chloride hexahydrate (SmCl_3_·6H_2_O, 99.9%), yttrium­(III) chloride hexahydrate (YCl_3_·6H_2_O, 99.9%) were supplied by Stream. Sodium alginate was purchased from ACROS. Calcium chloride (dried, powder, 97%), cobalt­(II) chloride hexahydrate (ACS, 98.0–102.0%), copper­(II) chloride dihydrate (ACS, 99 + %), nickel­(II) chloride hexahydrate (98%), zinc chloride (98 + %), iron­(II) chloride tetrahydrate (98%), l-cysteine (98 + %), d-cysteine (99%), o-vanillin (99%) and iron­(III) chloride hexahydrate (ACS, 97.0–102.0%) were purchased from Alfa Aesar. The ligand syntheses were carried out following modified literature procedures. 10.1016/j.molstruc.2017.10.090 Potassium chloride, magnesium chloride, and aluminum chloride were purchased from Sigma-Aldrich. All experiments were carried out with Milli-Q water.

### 2.2 Characterization Techniques

^1^H nuclear magnetic resonance (NMR) spectra were recorded on a Varian Mercury-400 (400 MHz) spectrometer. Crystal structures determined by single-crystal X-ray diffraction (SC-XRD) were visualized and graphically represented using Mercury. The Fourier-transform infrared spectroscopy (FTIR) was conducted using PerkinElmer Spectrum Two. A scanning electron microscope (SEM) was used on TM-3000 (Hitachi). An accelerating voltage of 15 kV was used for SEM. The SEM images were analyzed using ImageJ software. Mechanical properties were tested using a Materials Testing System (AGS-X-table type, Shimadzu) equipped with a 10 N load cell. The rheological characteristics of hydrogels were measured using a rheometer (AR 2000EX, TA Instruments) equipped with a 20 mm parallel plate. In the oscillation strain sweep, the strain was set from 0.1 to 1000% strain at 1 Hz under 25 °C with 10 points per decade. The luminescence apparatus used for PLQY measurements in our study is the LSLS-QY system (Model LSLS-QY, Serial Number 0033), provided by LiveStrong Optoelectronics Co., Ltd. This system is equipped with a 360 nm laser source (maximum power of 50 mW, stability <3% RMS over 4 h), integrated with a spectrometer (LS1700 model, wavelength range 300–1100 nm, resolution ∼ 1.5 nm, signal-to-noise ratio 2000:1) and an integrating sphere with an internal diameter of 10 cm. The measurement parameters, including integration time, averaging, and spectral range, were precisely controlled using dedicated LSLS-QY software provided by the manufacturer. Time-resolved photoluminescence (TRPL) measurements were performed using an Edinburgh Instruments FS5 spectrofluorometer equipped with a 405 nm pulsed solid-state laser diode as the excitation source. The laser operated with a pulse width of 1 μs and a repetition rate of 1 MHz. The emitted photoluminescence was collected in an epifluorescence configuration, passed through a monochromator, and detected by a photomultiplier tube (PMT). The TRPL decay profiles were recorded with a time step of 0.24 ns over a scan range of 0–500 ns. Each decay curve was deconvoluted with the instrument response function (IRF) to extract accurate carrier lifetimes. The FS5 system provides a temporal resolution of approximately 200 ps, and the measured IRF under our experimental configuration was about 250 ps. Zeta potential was measured by a Malvern Zetasizer Nano with a 633 nm wavelength laser. Inductively coupled plasma mass spectrometry (ICP–MS) was performed using a PerkinElmer ELAN DRC II instrument, and the discriminant analysis (LDA) was conducted with XLSTAT software.

### 2.3 X-ray Crystallographic Studies

Suitable crystals of complex **1** were mounted on glass fibers with perfluoropolyether oil and rapidly cooled in a stream of cold nitrogen gas to collect diffraction data at 150 K with the Bruker APEX2 diffractometer, and intensity data were collected with a combination of ϕ and ωscans. All data were corrected for Lorentz and polarization effects, and the APEX2 program in SADABS was used for absorption correction. The determination of the space group was based on a check of Laue symmetry and systematic absences and was verified using the structure solution. The structure was solved and refined using the SHELXTL package. All non-H atoms were localized using successive Fourier maps, and the hydrogen atoms were treated as riding models for their C atoms. Anisotropic thermal parameters were used for all non-H atoms, while fixed isotropic parameters were used for the H atoms. The molecular structure was drawn using the Oak Ridge Thermal Ellipsoid Plot (ORTEP).

### 2.4 Syntheses of Ligand and Complexes

#### (2*S*,4*R*)-2-(2-Hydroxy-3-methoxyphenyl)­thiazolidine-4-carboxylic Acid (L^SR^/L^RR^)

The thiazolidine-4-carboxylate^30^ and (2*S*,4*R*)-2-(2-hydroxy-3-methoxyphenyl) thiazolidine-4-carboxylic acid (L^SR^/L^RR^)^31^ ligands were synthesized according the reported literature. The methanol/water (10 mL/5 mL) solution containing l-cysteine (0.001 mol, 0.121 g) was mixed with a solution of o-vanillin (0.001 mol, 0.152 g) in methanol (10 mL). After stirred 6 h, the product, a white powder, was filtered, washed with methanol and dried in vacuo. Yield = 66%. NMR (DMSO-*d*_6_, 400 MHz): 7.04–6.07­(m, 3H), 5.86, 5.66­(2s, 1H, 2-H), 4.72–4.18, 3.84–3.80 (2t, 1H, 2-H), 3.77­(s, 3H), 2.93–3.35­(m, 2H). Select IR data (cm^–1^): 1637.72­(m), 1607.21­(w), 1589.39­(w), 1485.11­(m), 1448.86­(w), 1440.10­(m), 1345.15(s), 1279.53(s), 1241.28­(w), 1197.72­(w), 1183.34­(w), 1158.94­(w), 1080.62­(w), 1059.18­(m), 1028.18­(m), 983.62­(w), 936.40­(w), 926.14­(m), 837.93­(m), 804.70(s), 768.41(s), 739.52­(w), 710.26­(m), 677.24­(m), 656.51­(w).

#### (2*R*,4*S*)-2-(2-Hydroxy-3-methoxyphenyl)­thiazolidine-4-carboxylic Acid (L^RS^/L^SS^)

The methanol/water (10 mL/5 mL) solution, containing d-cysteine (0.001 mol, 0.121 g), was mixed with a solution of o-vanillin (0.001 mol, 0.152 g) in methanol (10 mL). After being stirred for 6 h, the product, a white powder, was filtered, washed with methanol, and dried in vacuo. Yield = 66%. NMR (DMSO-*d*_6_, 400 MHz): 7.04–6.07­(m, 3H), 5.85, 5.66­(2s, 1H, 2-H), 4.72–4.18, 3.84–3.80 (2t, 1H, 2-H), 3.77­(s, 3H), 2.9–3.35­(m, 2H). Select IR data (cm^–1^): 1633.84­(m), 1606.94­(m), 1588.94­(w), 1484.62(s), 1449.31­(w), 1405.89­(w), 1344.56(s), 1275.68(s), 1241.13(s), 1138.10­(m), 1158.66­(m), 1080.98­(m), 1058.98­(m), 1032.37­(m), 938.57­(w), 936.49­(w), 925.74­(m), 879.81­(w), 838.62­(m), 804.81(s), 768.41­(m), 739.29­(m), 710.53­(w), 678.27­(m), 656.55­(m).

### 2.5 Synthesis of [Ln_2_(*R*-tac)_4_(H_2_O)_8_]·6Cl (Ln = Tb(1); Eu(2); Sm(3))

To a solution of LnCl_3_·6H_2_O (0.125 mmol, 0.0332 g **(1)**, 0.0458g **(2)**, and 0.0456 g **(3)** in EtOH (5 mL) was added to a solution of L^SR^/L^RR^ (0.1875 mmol, 0.0479 g) in acetone (25 mL). The solution was stirred for 2 min and then filtered. The resulting colorless solution yielded rectangular, colorless crystals after 3 days. Yield: (38.4% **(1)**, 31.5% **(2)**, and 21.8% **(3)**).

#### 2.5.1 [Tb_2_(*R*-tac)_4_(H_2_O)_8_]·6Cl

Select IR data (cm^–1^): 1688.48 (s), 1632.83 (m), 1581.71 (m), 1434.86 (m), 1416.91 (s), 1388.85 (m), 1376.32 (m), 1229.79 (w), 1167.49 (m), 1041.36 (m), 840.57 (m), 666.85 (m). Anal. Calcd for C_34_H_86_Cl_6_N_4_O_23_S_4_Tb_2_ (**1·3C**_**2**_ **H**_**6**_ **O·4C**_**3**_ **H**_**6**_ **O**): C, 25.88; H, 5.49; N, 3.55; S, 8.13. Found: C, 25.91; H, 5.28; N, 3.68; S, 7.82. CCDC number: 2487027

#### 2.5.2 [Eu_2_(*R*-tac)_4_(H_2_O)_8_]·6Cl

Select IR data (cm^–1^): 1667.18 (s), 1614.49 (m), 1548.49 (m), 1432.19 (s), 1410.80 (m), 1377.02 (m), 1333.32 (m), 1273.32 (w), 1169.67 (m), 1049.19 (w), 661.46 (m), 628.82 (m). Anal. Calcd for C_31_H_74_Cl_6_N_4_O_21_S_4_Eu_2_ (**2·5C**_**3**_ **H**_**6**_ **O**): C, 25.09; H, 5.03; N, 3.78; S, 8.64. Found: C, 24.96; H, 4.69; N, 4.16; S, 8.59. CCDC number: 2486832

#### 2.5.3 [Sm_2_(*R*-tac)_4_(H_2_O)_8_]·6Cl

Select IR data (cm^–1^): 1682.86 (s), 1614.70 (m), 1547.88 (m), 1428.90 (m), 1409.15 (m), 1376.59 (m), 1332.77 (m), 1273.18 (w), 1169.76 (m), 1050.71 (m), 661.09 (m), 601.25 (m). Anal. Calcd For C_27_H_68_Cl_6_N_4_O_20_S_4_Sm_2_ (**3·1C**_**2**_ **H**_**6**_ **O·3C**_**3**_ **H**_**6**_ **O**): C, 22.99; H, 4.86; N, 3.97; S, 9.09. Found: C, 22.62; H, 4.68; N, 4.24; S, 9.28.

### 2.6 Preparation of Hydrogels

The Ln complex (i.e., Tb, Eu, and Sm complex) and the sodium alginate were dispersed separately in distilled water at concentrations of 1.5 and 10 wt %, respectively. The two solutions were mixed at equal volume through a dual-channel syringe and stored at 25 °C for 24 h to ensure proper gel formation before further use.

### 2.7 Microstructures of Hydrogels

The hydrogels were prepared and subjected to lyophilization using a freeze-dryer (UNISS FDM-2) under conditions of −80 °C and 10 mTorr. The resulting lyophilized hydrogel samples were then examined for their microstructure using a Hitachi TM-3000 tabletop scanning electron microscope (SEM). The pore size analysis of the lyophilized hydrogel was measured by mercury porosimeter (micromeritics AutoPore IV 9520). The experiments were performed at low pressure (345 kPa) and at high pressure (414 MPa). This method was used to obtain information on pores larger than 3.6 nm. Micro-CT was employed to investigate the internal porous structure of the hydrogels. A 200 μL sample of fully lyophilized hydrogel was subjected to X-ray micro-CT imaging (Skyscan 1076). This technique allowed for a detailed, slice-by-slice analysis of the hydrogel’s microstructure.

### 2.8 Rheological and Mechanical Measurements of Hydrogels

The rheological properties of hydrogels were carried out by a rheometer with a 20 mm parallel plate. In the oscillation strain sweep test, the strain was swept from 0.1 to 1000% strain for 1 Hz at 25 °C with 10 points per decade. In the continuous flow sweep test, the shear rate was set between 0.1 and 100 s^–1^ for 1 Hz at 25 °C with 10 points per decade, illustrating the shear thinning property. The self-healing property was demonstrated by the cyclic strain time sweep test under the low strain of 1.0% and high strain of 500% alternatively.

Cylindrical hydrogel samples (diameter = 8 mm, height = 7 mm) were tested under uniaxial compression at a constant speed of 1 mm min^–1^. The samples were compressed up to approximately 80–100% strain, depending on the formulation. The compressive modulus was calculated through linear fitting of the data between 10% and 20% strain, where elastic behavior was observed.

### 2.9 Swelling, Degradation, and Thermal Stability of Hydrogels

The hydrogels were prepared freshly, and their weights (w_1_) were recorded. Subsequently, the hydrogels were immersed in DI water. To ensure consistent swelling, the hydrogels were placed in a precisely controlled incubator set at a temperature of 37 °C and a shaking speed of 100 rpm. After treatment, the hydrogels were carefully extracted from the DI water. The excess liquid on the hydrogel surface was carefully wiped away to ensure accurate measurements, and then it was weighed (w_2_). The swelling ratio of the hydrogels was calculated using the following equation.

$$\mathrm{Swelling}\;\mathrm{ratio}=w_{2}/w_{1}$$

$$\mathrm{Water}\;\mathrm{content}\left(\%\right)=\left[\left(w_{2}-w_{1}\right)/w_{2}\right]\times 100$$

The degradability of hydrogels was determined by immersing the lyophilized hydrogels (∼19 mg, w_0_) in DI water. (1.0 mL), and then the hydrogels were lyophilized to measure mass (w_D_) after immersion.

$$\mathrm{Weight}\;\mathrm{remaining}\;\mathrm{ratio}\left(\%\right)=\left(w_{D}/w_{0}\right)\times 100$$

Differential scanning calorimetry (DSC) was performed with a TA Instruments Q-20 to evaluate the thermal properties of the hydrogels. The analysis was performed at a heating rate of 10 °C per minute in a temperature range from −30 to 200 °C under a nitrogen flow of 50 mL/min.

### 2.10 Metal Ion Sensing Using Alg-Eu Lyophilized Hydrogels

The Alg-Eu hydrogels were prepared with a defined volume (100 μL) and then lyophilized before immersing in the metal ion solutions (1 mL) for sensing. The lyophilized Alg-Eu hydrogels were soaked separately into various metal ion solutions (Na^+^, K^+^, Cu^2+^, Ca^2+^, Co^2+^, Ni^2+^, Mg^2+^, Zn^2+^, Fe^2+^, Fe^3+^, and Al^3+^) with 10^–3^ M for 1 h at room temperature. The luminescence spectra of the hydrogels were carried out on photoluminescence and efficiency measurement system (LSLS-QY, LiveStrong Optoelectronics) with an integrating sphere and an excitation at 360 nm. To establish a hydrogel-based analytical strategy, various concentrations of Cu^2+^ ions (2–10 μM) were prepared, and then Alg-Eu lyophilized hydrogels were treated with the concentrations of the Cu^2+^ ions for 1 h at room temperature. The luminescence changes of Alg-Eu lyophilized hydrogels were determined for analysis.

### 2.11 Statistical Analysis

All measurements were repeated three times, and the error bars in the figures represent the standard deviation. A *t*-test was used to analyze whether the differences between the data were statistically significant. Significance was set at *p* < 0.05 with *, **, or *** indicating *p* < 0.05, 0.01, or 0.001, respectively.

## 3 Results and Discussions

### 3.1 Syntheses and Characterizations of Ligand and Precoordinated Ln Complexes

Polydentate heterocyclic thiazolidine ligands, synthesized from aldehydes and amino acids, have been widely utilized in the synthesis of lanthanide clusters,^32–34^ where the ligand provides both oxygen (O)- and nitrogen (N)-based chelating sites for lanthanide complex formation. In particular, aldehydes and cysteine can be used for ligand synthesis, as amino thiols generated heterocyclic thiazolidines and created a novel chiral center.^35,36^ In this study, two stereoisomers, ((2*S*,4*R*)-2-(2-hydroxy-3-methoxyphenyl) thiazolidine-4-carboxylic acid (L^SR^/L^RR^)) synthesized by o-vanillin and cysteine, were used as ligands for lanthanide complexation (Scheme S1). Complexes **1**–**3** were synthesized by reacting LnCl_3_·6H_2_O (Ln = Tb (**1**), Eu (**2**), and Sm (**3**)) with L^SR^/L^RR^ in a metal-to-ligand ratio of 2:3 in an EtOH/acetone mixture (Scheme S1). (R)-thiazolidine-4-carboxylic acid (R-tac) was formed in situ due to the cleavage of C–C bond of ligand,^37,38^ with the proposed mechanism shown in Scheme S2. R-tac could be obtained by organic synthesis, while no product will be obtained due to the solubility issue of R-tac. The ^1^H NMR spectra of L^SR^/L^RR^ and R-tac were shown in Figures S1 and S2, respectively.

Complex **1** (i.e., **Tb**_**2**_ **(** ***R*** **-tac)**_**4**_ **(H**_**2**_ **O)**_**8**_ **·6Cl**) crystallized in space group *P*4_3_2_1_2 of cubic system. As shown in Figure 1a, Tb1 in complex **1** was located in the center of a heptagonal pyramid geometry, while Tb2 was situated in the center of an octagonal geometry, which was determined by **SHAPE 2.1** (Table S1).^39–42^ Each Tb central is octa-coordinated by four equivalent water molecules and four oxygen atoms from four *R*-tac in an unidentate mode. Tb^3+^ centers were bridged by four carboxylate groups in a μ_2_:η^1^:η^1^ fashion. The Cl^–^ anions were hydrogen-bonded to water molecules and the nitrogen atom in the thiazolidine ring of the R-tac ligands. The Tb···Tb distance was 4.4947(9) Å. Tb–O bond lengths ranged from 2.261(8) to 2.457(11) Å. The selected bond lengths and angles are shown in Tables S2–S6. Powder X-ray diffraction (PXRD) measurements showed that the pattern of complex **1** closely matched the simulated result. In comparison, the PXRD patterns of complexes **2** and **3** exhibited slight differences, likely due to the absence of single crystals for these complexes (Figure 1b).

**Figure 1:**
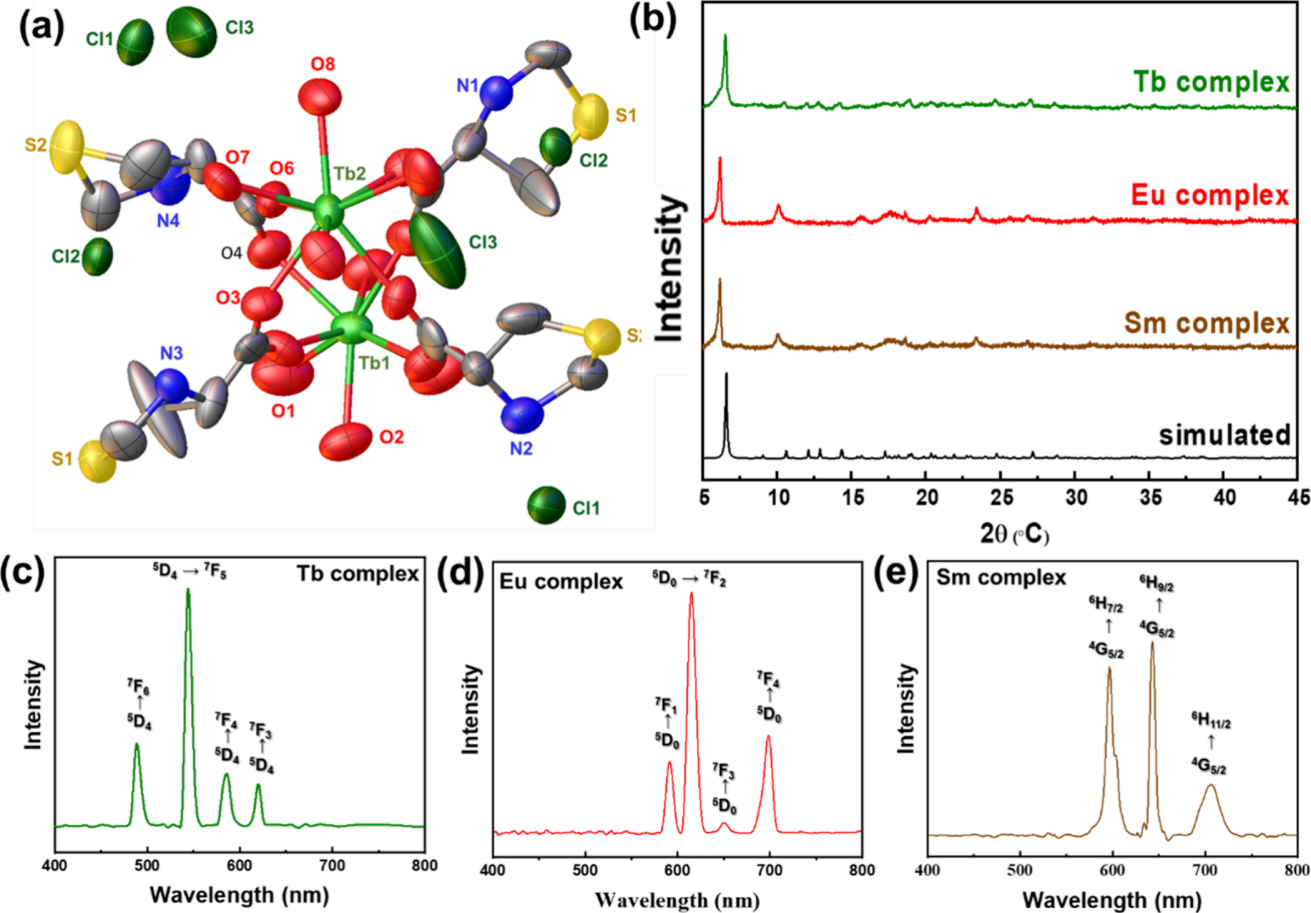
(a) Crystal structures of [Tb_2_(*R*-tac)_4_(H_2_O)_8_]·6Cl (**1**). H atoms are omitted for clarity. Color code: Dark green (Tb), blue (N), red (O), yellow (S), and green (Cl). (b) PXRD patterns of Ln complexes. The simulated PXRD pattern was generated based on the crystal structure of the Tb complex. Luminescence spectra of (c) Tb, (d) Eu, and (e) Sm complexes.

The precoordinated Ln complexes were characterized through several techniques (i.e., Fourier-transform infrared spectroscopy (FTIR), excitation spectroscopy, photoluminescence spectroscopy, scanning electron microscope (SEM), and zeta potential) to reveal their chemical and physical properties. In the FTIR spectrum of the Eu complex, the asymmetric carboxylate vibrational band shifted from 1638 to 1689 cm^–1^, indicating that the ligand was successfully coordinated with the Eu^3+^ ions (Figure S3). Similarly, the FTIR spectra of the Tb and Sm complexes exhibited distinct bands corresponding to asymmetric carboxylate vibrations at 1690 and 1685 cm^–1^, respectively (Figure S3). The excitation spectra of the Ln complexes are shown in Figure S4. All samples exhibited broad excitation bands in the range of 250–400 nm, which could be attributed to the thiazolidine-based ligands. These absorption bands served as weak chromophores capable of partially transferring energy to the Ln^3+^ centers through ligand-to-metal charge transfer (LMCT) processes. This sensitization was less efficient than in classical antenna systems, likely due to the presence of coordinated solvent molecules that further quenched the luminescence.

Luminescence spectra were recorded under identical excitation conditions to isolate the influence of the lanthanide ions on the photoluminescence characteristics of Ln complexes. Tb complex emitted mainly at 489, 543, 588, and 620 nm, correlating with the ^5^D_4_→^7^F_J_ transitions (J = 6, 5, 4, and 3), with the ^5^D_4_→^7^F_5_ transition being the most prominent for green luminescence (Figure 1c). Eu complex featured peaks at 591, 652, and 702 nm corresponded to the ^5^D_0_→^7^F_n_ transitions (n = 1, 3, and 4), and also showed a red luminescence peak at 616 nm due to the electronic transition ^5^D_0_→^7^F_2_ (Figure 1d). Sm complex exhibited three main luminescence peaks, each associated with specific transitions from ^4^G_5/2_ to ^6^H_n_ (n = 7/2, 9/2, and 11/2) (Figure 1e). The photoluminescence quantum yields (PLQYs) of the Tb, Eu, and Sm complexes were also measured with the excitation wavelength of 360 nm, showing 0.17%, 2.22%, and 0.10%, respectively. Also, time-resolved photoluminescence (TRPL) measurements revealed the average lifetimes of Eu, Tb, and Sm complexes were 7.41, 4.74, and 1.63 ns, respectively (Figure S5). The lifetimes of the complexes were correlated to their PLQY values, indicating that the luminescence efficiency is lifetime-dependent.

Sm^3+^ generally exhibits weaker luminescence than Eu^3+^ and Tb^3+^ due to the smaller energy gap between the emitting level (^4^G_5_/_2_) and lower-lying states, which facilitates nonradiative relaxation according to the energy gap law.^43^ In addition, Sm^3+^ emission is more susceptible to vibrational quenching by O–H oscillators in hydrated environments such as alginate hydrogels, further reducing the observed emission intensity.^44,45^

The Tb, Eu, and Sm complexes exhibited similar low water solubility without obvious differences in dispersion state (Figure S6). SEM was employed to examine the morphology of the Ln complexes. However, the SEM images did not reveal significant differences in particle size among the Eu, Tb, and Sm complexes, as the observed structures were primarily bulk aggregates (Figure S7). Besides, the zeta potentials of the Tb, Eu, and Sm complexes were approximately 8.96, 8.65, and 8.91 mV, respectively (Figure S8). These positive values indicated that the NH_2_^+^ of the coordinating ligand conferred a net positive charge to the complex surface.

It should be noted that the compounds reported in this work are molecular coordination complexes that crystallize as single crystals suitable for single-crystal X-ray diffraction analysis. Therefore, they are fundamentally different from nanoparticle or nanocrystal systems, in which particle-size characterization (e.g., TEM or DLS) is typically required. In this study, the crystals were obtained from solutions as single molecular crystals with micrometer-scale size. Accordingly, the structural information on the complexes was determined by single-crystal X-ray diffraction, which provides precise structural characterization of the molecule (Figure S9).

### 3.2 Formation and Characterization of Hydrogels

Three Ln complexes (i.e., Tb, Eu, and Sm complexes) were mixed with alginate using a dual-channel syringe to fabricate Alg-Ln hydrogels through electrostatic interactions. The ^1^H nuclear magnetic resonance (NMR) spectroscopy was used to characterize the structure of alginate (Figure S10), and the weight-average molecular weight (*M*_w_) of alginate was 157 kDa (dispersity (Đ = 1.06) determined by gel permeation chromatography (GPC).

To support the proposed cross-linking mechanism, we compared the FT-IR and XPS spectra of hydrogels cross-linked by precoordinated lanthanide complexes (Alg-Ln) with those cross-linked by lanthanide salts (Alg-LnN). In the FT-IR spectra, pristine alginate exhibited characteristic asymmetric and symmetric carboxylate stretching vibrations at 1627 and 1412 cm^–1^, respectively (Figure S3). After hydrogel formation, these bands shifted due to interactions between alginate carboxylate groups and lanthanide-containing cross-linkers. For the Alg-Ln hydrogels, the carboxylate bands shifted to higher wavenumbers (e.g., 1655 and 1457 cm^–1^ in Alg-Eu) (Figure S3), indicating electrostatic interactions between alginate chains and the precoordinated Ln complexes.^46,47^ The Alg-LnN hydrogels (Alg-EuN, Alg-TbN, and Alg-SmN) exhibited similar spectral changes, with the carboxylate bands appearing at 1595–1621 cm^–1^ and 1416–1419 cm^–1^ (Figure S11). These shifts were attributed to direct coordination between free Ln^3+^ ions and alginate carboxylate groups, consistent with the conventional ion-cross-linked structure of alginate hydrogels.^48,49^

On the other hand, XPS was further applied to examine the stability of the lanthanide complex in the hydrogel (Figure S12). It could be hypothesized that if the Eu complex were fully dissolved in the hydrogel and released as free Eu^3+^ ions, the interaction in Alg-Eu would be expected to resemble that of Alg-EuN, where Eu^3+^ directly coordinated with the carboxylate groups of alginate. Here, the XPS results revealed distinct spectral features between the two Alg-Eu and Alg-EuN hydrogel systems. For the precoordinated system (Alg-Eu), the C–N peak in the C 1s spectrum shifted from 285.16 eV in the Eu complex to 286.34 eV (Figures S12c,l), indicating a change in the electronic environment due to electrostatic interactions between the C–N groups and the – COO^–^ groups of alginate, which also showed that the Eu complex still participated in the hydrogel network through electrostatic interactions with alginate. By contrast, for the lanthanide ion system (Alg-EuN), the O–C = O peak in the O 1s spectrum shifted from 532.53 to 533.19 eV relative to pristine alginate (Figures S12e,h), which was consistent with direct coordination between Eu^3+^ ions and alginate carboxylate groups. These distinct XPS behaviors suggested that the interaction mode in Alg-Eu was different from that in Alg-EuN, demonstrating that the precoordinated Eu complex was not simply converted into free Eu^3+^ ions in the hydrogel. Instead, the complex remained involved in network formation through complex-mediated interactions.

The formation of Alg-Ln hydrogels was investigated on a macroscale as well as rheological analysis. The gel-like Alg-Ln hydrogels were observed in the macroscale as well as under oscillating time sweeps, showing that the storage modulus (*G’*) was higher than the loss modulus (*G’’*) at continuous time scans compared to the liquid-like behavior of alginate and Ln complexes (Figure S13).^49^The rheological properties of Alg-Ln hydrogels were dose-dependent. For example, *G’* values of Alg-Eu hydrogels at Eu complex concentrations of 0.25, 0.5, 0.75, and 1.0 wt % were ∼ 4500, 4890, 8820, and 24470 Pa, respectively (Figure S14). In addition, the gelation times of the Alg-Ln hydrogels were compared by oscillating time sweeps, where the gel point was determined by the crossing point of *G’* and *G’’* (Figure 2a). The gelation times of Alg-Tb, Alg-Eu, and Alg-Sm hydrogels were ∼ 19, 16, and 15 min, respectively, indicating that Alg-Sm exhibited the fastest gelation (Figure 2b). In general, the accelerated gelation of hydrogels can be attributed to several factors, including the structure of the ligand^50^ and the radius of the metal ion.^29,51,52^ Here, the ionic radius of Sm^3+^ (r= 109.8 pm) is larger than that of Eu^3+^ (r= 108.7 pm) and Tb^3+^ (r= 106.3 pm). Therefore, the rapid gelation in the Alg-Sm hydrogel was likely due to the larger volume of the Sm complex, which created more space between alginate chains and promoted electrostatic cross-linking within the hydrogel network more effectively than the smaller Eu and Tb complexes. It should be noted that the *G’* values shown in Figure 2a represent the early stage gelation kinetics, where all Alg–Ln hydrogels were prepared using 0.75 wt % Ln complexes and *G’* was monitored continuously during network formation. Under these identical conditions, the final *G’* values appeared similar because this measurement captured the gelation process rather than the fully developed network structure.

**Figure 2:**
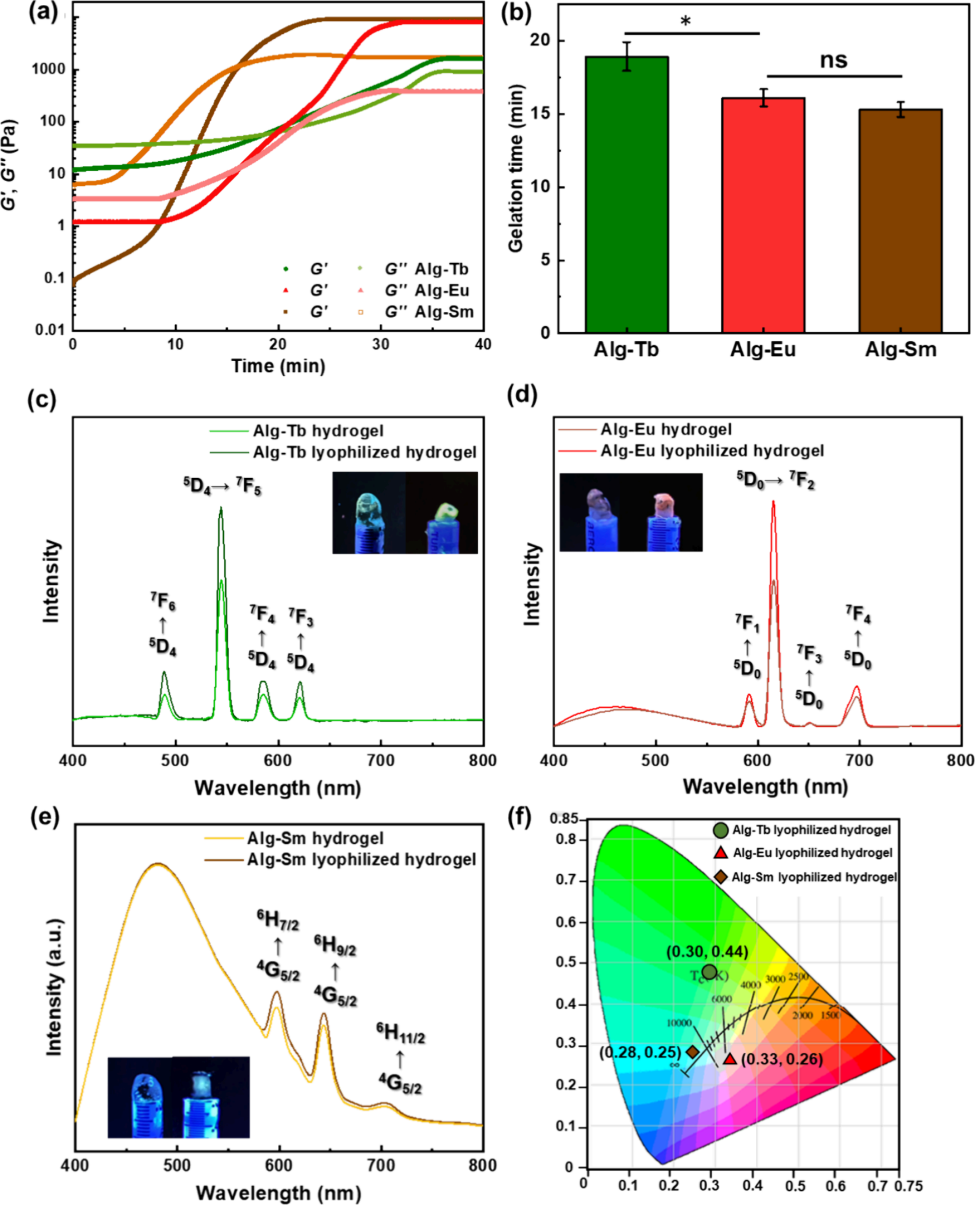
(a) Continuous time sweeps and (b) gelation times of Alg-Ln hydrogels. Alg-Ln hydrogels were prepared with alginate (5 wt %) and Ln complex (0.75 wt %). Luminescence spectra of (c) Alg-Tb, (d) Alg-Eu, and (e) Alg-Sm hydrogel and lyophilized hydrogels. (λ_ex_ = 360 nm) Inset: images of hydrogel (left) and lyophilized hydrogel (right) under UV light (365 nm). (f) CIE chromaticity coordinates of Alg-Tb, Alg-Eu, and Alg-Sm. Alg-Ln hydrogels were prepared with alginate (5 wt %) and Ln complex (0.75 wt %). *Statistical significance was set at *p* < 0.05 (*p* < 0.05).

The luminescence spectra of the Alg-Ln hydrogels with hydrated and lyophilized forms were recorded and further converted to Commission Internationale de l’Éclairage (CIE) coordinates. The luminescence spectrum of the Alg-Tb hydrogel showed four characteristic peaks at 488, 543, 583, and 621 nm, indicating transitions from the ^5^D_4_ to the ^7^F_j_ level (*J* = 6–3) (Figure 2c). The luminescence spectrum of the Alg-Eu hydrogel showed distinct peaks at around 591, 616, 650, and 702 nm, corresponding to the ^5^D_0_ → ^7^F_j_ (*J* = 0–4) transitions of Eu^3+^ ions, along with a broad peak in the 400–500 nm range attributed to the alginate polymer itself (Figure 2d and Figure S15). In addition, the Alg-Sm hydrogel exhibited three primary emission peaks at 561, 596, and 642 nm, each corresponding to specific transitions from 4G_5/2_ to the corresponding ^6^H_J_ (*J* = 5/2, 7/2, and 9/2) states (Figure 2e). The luminescence intensity of the Alg-Ln lyophilized hydrogels can be further modulated by changing the amount of Ln complexes, with 1 wt % of Ln complexes providing the highest luminescence in the hydrogel network (Figure S16).

The resulting CIE color values closely matched the visual observations under UV irradiation. The Alg-Eu hydrogel exhibited distinct red luminescence (Figure 2f), while the Alg-Tb hydrogel emitted a vivid green luminescence (Figure 2f). In contrast, Alg-Sm hydrogel showed only weak blue fluorescence (Figure 2f), which was primarily due to intrinsic emission from the alginate matrix and not from the incorporated Sm complex. To compare the luminescence of the Alg-Ln hydrogels quantitatively, the photoluminescence quantum yield (PLQY) of Alg-Ln hydrogels was calculated according to eq 1:

$$\mathrm{PLQY}=\frac{\mathrm{The}\;\mathrm{number}\;\mathrm{of}\;\mathrm{photons}\;\mathrm{emitted}}{\mathrm{The}\;\mathrm{number}\;\mathrm{of}\;\mathrm{photons}\;\mathrm{absorbed}}=\frac{E_{c}-E_{a}}{L_{a}-L_{c}}$$ 1

Where *E*_a_ and E_c_ were the integrated luminescence measured from empty and with the sample in the integrating sphere under 360 nm excitation. L_a_ and L_c_ were the integrated excitation profiles with and without the sample, respectively, when directly excited by the incident beam.

The PLQY values of Alg-Tb, Alg-Eu, and Alg-Sm hydrogels were 0.17 ± 0.06%, 2.01 ± 0.07%, and 0.06 ± 0.01%, respectively (Table S7). On the other hand, the PLQY values of Alg-Tb, Alg-Eu, and Alg-Sm lyophilized hydrogels were 0.20 ± 0.03%, 2.51 ± 0.06%, and 0.07 ± 0.02%, respectively. For the Alg-Tb and Alg-Sm systems, the differences between wet and lyophilized hydrogels fell within the experimental uncertainty, indicating that the observed variations were not statistically significant. In contrast, the Alg-Eu system showed an increase in PLQY after lyophilization (∼125%), suggesting a meaningful enhancement.

This phenomenon can be attributed to the coordination of water molecules to the Ln ions within the hydrogel structure, whose O–H overtones promote nonradiative relaxation pathways and effectively quench the luminescence of the excited Ln ion.^26,27^ Compared with hydrated samples, the lyophilized hydrogels exhibited more than a 10% increase in PLQY. Furthermore, the preservation of key spectral features indicated the structural stability and robustness of the lanthanide coordination environment within the alginate matrix. The incorporation of Ln complexes into the alginate matrix mitigated interference from coordinated water and maintained appropriate luminescence properties.

The emission origin in the Alg-Sm hydrogel was further clarified by comparison with the calcium (Ca) ion-cross-linked alginate (Alg-CaN) system (Figure S17). The CIE chromaticity coordinate of the Alg-CaN hydrogel was in the blue region, indicating that alginate itself exhibited blue fluorescence. Notably, the emission profile of the Alg-Sm hydrogel closely resembled that of the Alg-CaN hydrogel, showing a similar broad emission band. Therefore, the dominant emission in the Alg-Sm hydrogel originated from the alginate matrix, while the contribution from Sm^3+^ was minimal.

As a control, Ln­(NO_3_)_3_ was used as an ionic cross-linker for alginate in hydrogel formation. However, when the same molar amount of Ln ions was used, the direct addition of Ln­(NO_3_)_3_ to alginate to form the Alg–LnN hydrogel did not produce mechanically stable, cylindrically shaped hydrogels, in contrast to the Alg–Ln hydrogel (Figure S18). The precoordinated complexes efficiently induced gelation, producing robust, free-standing hydrogel columns through enhanced electrostatic interactions and coordination with alginate chains, establishing more uniform network structures. Furthermore, the PLQY values of complex-cross-linked and ion-cross-linked lyophilized hydrogels were comparable (e.g., Alg-Tb= 0.20 ± 0.03% and Alg-TbN= 0.21 ± 0.01%) (Tables S7 and S8). Nevertheless, the enhanced structural integrity obtained using precoordinated Ln complexes is highly beneficial, as it allows the formation of mechanically robust alginate hydrogels suitable for practical handling, precise shaping, and potential incorporation into functional devices.^48,49^

### 3.3 Microstructures and Properties of Hydrogels

The porous microstructures of the Alg-Ln lyophilized hydrogels were visualized under SEM (Figure 3a), and the elemental mapping through energy dispersive X-ray spectroscopy (EDS) confirmed the homogeneous distribution of Ln ions in the Alg-Ln hydrogel matrix (Figure S19). Microcomputed tomography (micro-CT) and mercury intrusion porosimetry (MIP) were also employed to quantitatively analyze the pore size distribution and porosity of the Alg-Ln lyophilized hydrogels (Figure 3b–d). In the micro-CT analysis, the pore sizes of the Alg-Tb, Alg-Eu, and Alg-Sm lyophilized hydrogels were 116, 93, and 79 μm, respectively (Table S9). In the MIP analysis, the pore sizes of the Alg-Tb, Alg-Eu, and Alg-Sm lyophilized hydrogels were 52.2, 46.4, and 45.0 μm, respectively (Table S10). The pore diameter observed by micro-CT images is expected to be larger than that measured through MIP due to the differing measurement principles, size definitions, and sampled regions inherent to the two techniques.^53,54^ The results showed that the Alg-Sm lyophilized hydrogel possessed a smaller average pore diameter compared to the Alg-Eu and Alg-Tb lyophilized hydrogels. Remarkably, micro-CT analysis also showed that all Alg-Ln lyophilized hydrogels had a greater proportion of open porosity than closed porosity, with open porosity of ∼ 80%. Also, MIP measurement indicated that the porosity of the Alg-Ln lyophilized hydrogels was ∼ 98%. Therefore, the highly porous structural configuration of Alg-Ln lyophilized hydrogels may enhance the swelling ability of hydrogels, as open pores with open surfaces facilitate fluid flow and penetration.

**Figure 3:**
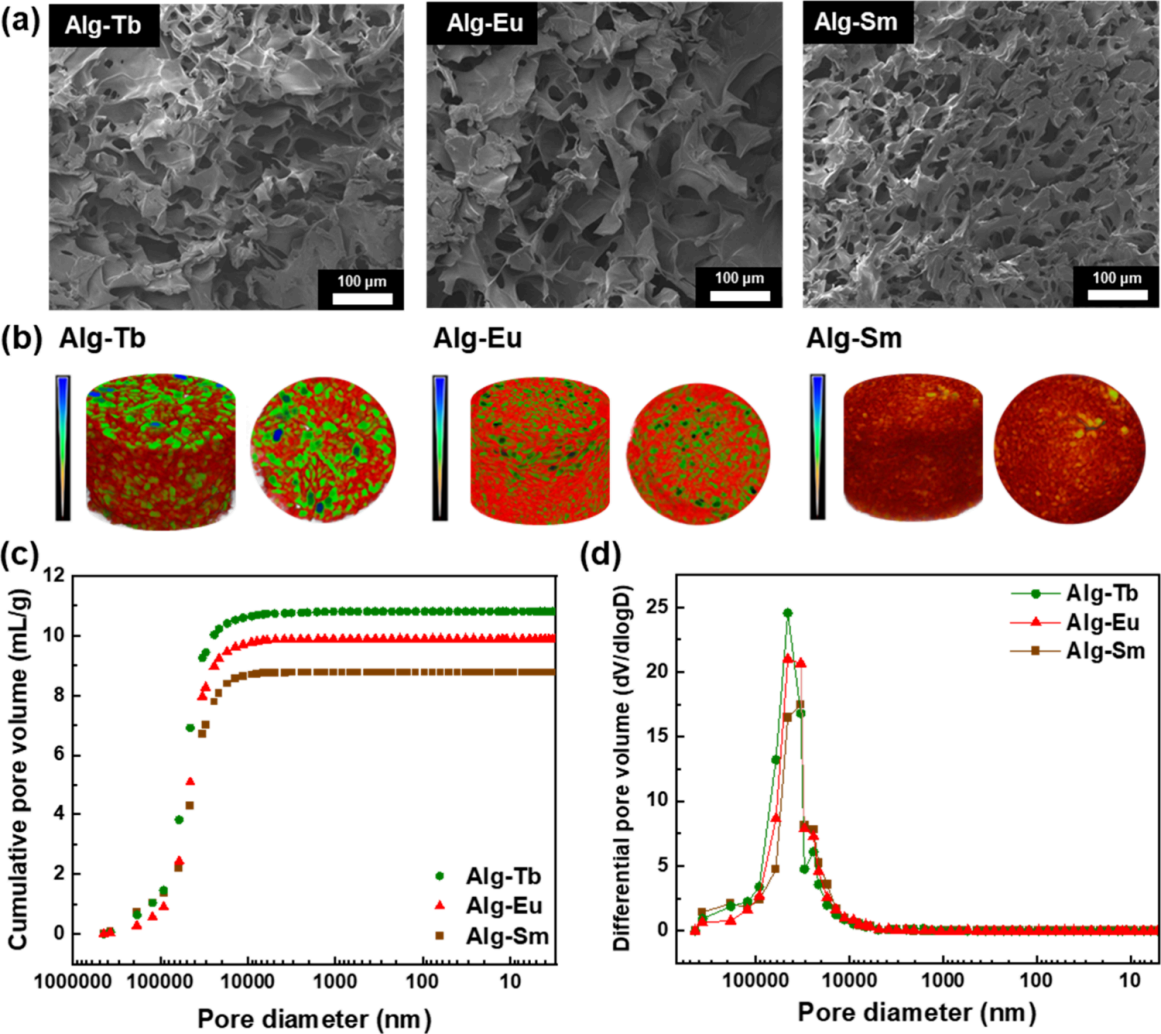
(a) Representative SEM images of Alg-Ln lyophilized hydrogels (with Ln complexes of 0.75 wt %). (b) Micro-CT images and cross-sectional views of Alg-Ln lyophilized hydrogels. The pore sizes of Alg-Ln lyophilized hydrogels were indicated by using a color scale, with higher values on the color bar exhibiting larger pore sizes. It should be noted that this scale represents relative measurements. Pore size distributions were presented as (c) cumulative pore volume and (d) differential pore volume (d*V*/d*l*ogD) of Alg-Ln lyophilized hydrogels.

The rheological behavior of the Alg–Ln hydrogels was systematically examined through oscillatory strain sweep measurements to elucidate the dose-dependent cross-linking effect of the precoordinated Ln complexes on alginate hydrogel formation (Figure S20). Alg-Ln hydrogels were prepared using alginate (5 wt %) and Ln complexes at different concentrations, ranging from 0.25 wt % to 1 wt %. The equilibrium rheological properties were obtained from oscillatory strain-sweep measurements on fully formed hydrogels after precursors were mixed for 24 h. The rheological properties of the three Alg-Ln hydrogels exhibited a clear dose-dependent response, with the *G’* values of the three Alg-Ln hydrogels increasing with the higher concentration of the Ln complexes. For example, *G’* values of the Alg-Tb hydrogels with Ln complexes of 0.25, 0.50, 0.75, and 1.0 wt % were 3706 ± 210, 4645 ± 396, 5212 ± 444, and 5708 ± 361 Pa, respectively (Table S11). On the other hand, the *G′* values of the three Alg–Ln hydrogels can be tuned by varying the type of ions present in the Ln complexes. With the same concentration of Ln complexes (1 wt %), the *G’* values of the Alg-Tb, Alg-Eu, and Alg-Sm hydrogels were 5708 ± 361, 24465 ± 2084, and 43623 ± 3601 Pa, respectively (Table S11). The cross-linking density of the Alg-Ln hydrogel network was further calculated using the theory of rubber elasticity: *G’* = *v*RT, where *G’* is the storage modulus in Pa, *v* is the cross-linking density in moles of elastically active network chains per cubic meter, R is the gas constant (8.314 J K^–1^ mol^–1^), and T is the temperature in Kelvin (298.15 K) (Figure 4a). For example, the cross-linking densities of the Alg-Tb, Alg-Eu, and Alg-Sm hydrogels (with 1 wt % Ln complex) were determined to be 2.3 ± 0.1, 9.9 ± 0.8, and 17.6 ± 1.5 mol/m^3^, respectively (Table S11). In addition, the flow points for the Alg-Tb, Alg-Eu, and Alg-Sm hydrogels (with 1 wt % Ln complex) were measured to be 15.8 ± 0.6%, 28.5 ± 1.3%, and 41.5 ± 2.3%, respectively (Table S11). Therefore, Alg-Sm hydrogel possessed higher cross-link density and flow point than Alg-Eu and Alg-Tb hydrogels. Stress relaxation experiments were performed to further evaluate the cross-linking behavior of the hydrogels. The three hydrogels exhibited distinct stress-relaxation behaviors: Alg-Sm showed the slowest stress relaxation, followed by Alg-Eu, whereas Alg-Tb relaxed more rapidly (Figure S21). The slower stress relaxation of Alg-Sm hydrogel indicated that the network structure was more constrained and that the effective cross-linking interactions within the hydrogel were stronger.

**Figure 4:**
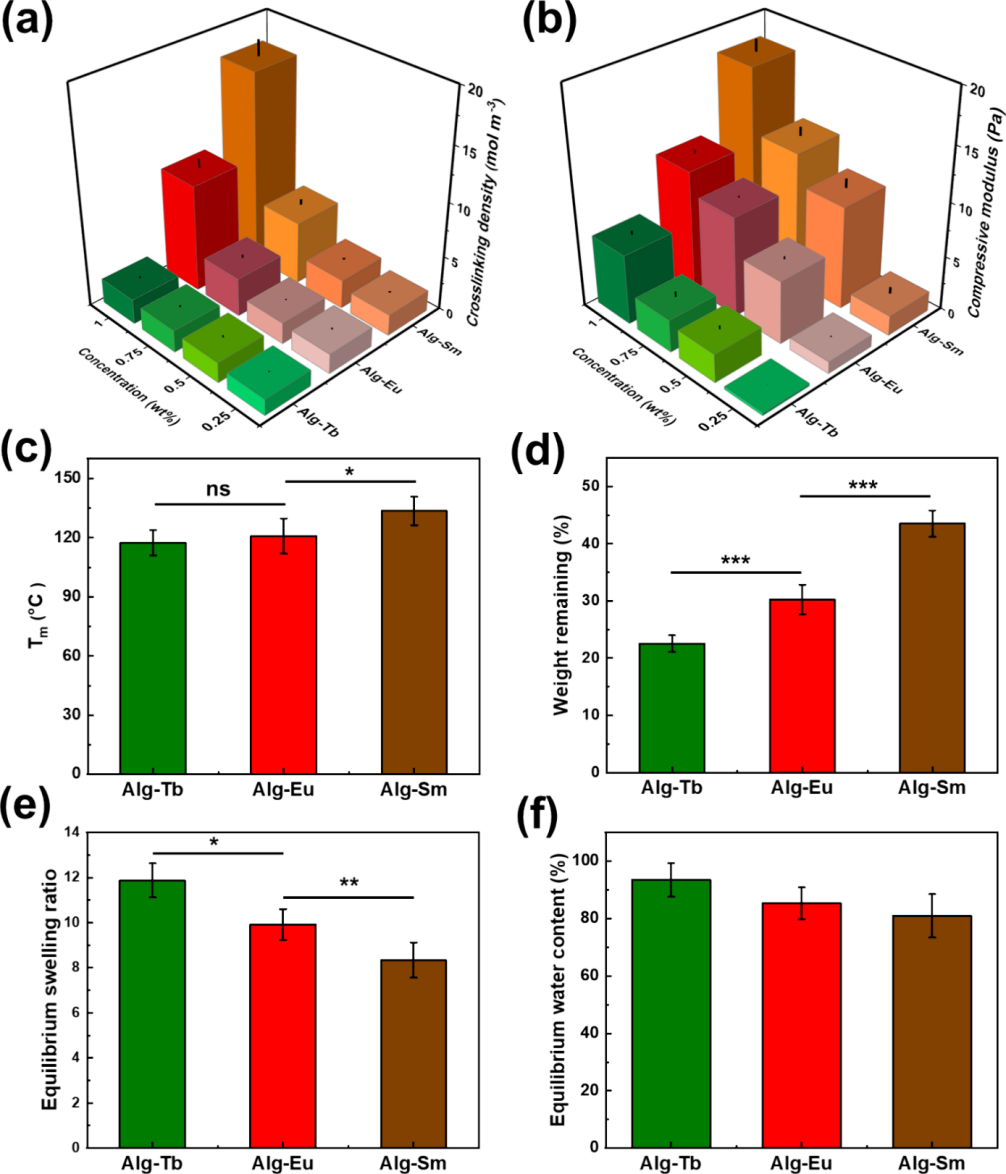
(a) Cross-linking densities and (b) compression moduli of Alg-Ln hydrogels. (c) *T*_m_ values of Alg-Ln hydrogels with 0.75 wt % Ln complex. (d) Weight remaining of Alg-Ln hydrogels with 0.75 wt % Ln complex after immersion for 12 days. (e) Equilibrium swelling ratio and (f) equilibrium water content of Alg-Ln hydrogels with 0.75 wt % Ln complex after immersion for 2 h. Significance was set at *p* < 0.05 with *, **, or *** indicating *p* < 0.05, 0.01, or 0.001, respectively.

Compression tests were also performed to further evaluate the mechanical properties of the Alg-Ln hydrogels, showing Alg-Sm hydrogel exhibited a higher mechanical strength (18.0 ± 1.1 kPa) than Alg-Tb (6.5 ± 0.3 kPa) and Alg-Eu (11.2 ± 0.3 kPa) hydrogels (Figure 4b and Figure S22). These results are consistent with the *G’* values observed in the rheological studies, further supporting the correlation between cross-link density and mechanical strength within the hydrogel network. It should be noted that the hydrogels did not exhibit catastrophic fracture but instead showed progressive densification under compression, a typical feature of highly hydrated alginate-based networks. The apparent “100% strain” in Figure S22 corresponded to near-complete geometric collapse of the hydrogel rather than true zero thickness, as the compliant gels flatten while retaining residual thickness between the plates. Also, the relatively low alginate concentration (5 wt %) was selected for the compression testing to ensure sufficient chain mobility and homogeneous mixing with the precoordinated lanthanide complexes. At higher alginate concentrations (>6–7 wt %), the viscosity of the precursor solution became too high to allow uniform mixing using the dual-syringe method, leading to inhomogeneous gelation and poor reproducibility. Therefore, processability and network uniformity, rather than solubility, were the primary limiting factors. Besides, the unusual stress–strain profiles observed for Alg–Sm hydrogels arose from their higher cross-linking density and more heterogeneous internal pore structure, which led to localized collapse of the porous network during compression.

The mechanical properties of alginate hydrogels cross-linked by different ions were further compared through rheology. Ca^2+^-cross-linked alginate (Alg-CaN) hydrogels exhibited a *G′* of ∼ 1.9 kPa, while alginate hydrogels directly cross-linked with lanthanide ions (Alg–LnN) displayed lower *G′* values ranging from 0.7 to 1.0 kPa (Figure S23). The results indicated that direct ionic cross-linking by Ln^3+^ ions alone did not provide stronger mechanical reinforcement than Ca^2+^. Ca^2+^ typically forms stronger alginate hydrogels because it fits well into the guluronate (G-block) cavities and forms well-organized “egg-box” junction zones, resulting in a more uniform and cooperative cross-linking network. However, lanthanide ions often exhibit higher coordination numbers and strong hydration shells, which can lead to less ordered coordination with alginate chains and more heterogeneous cross-linking domains. Consequently, the effective network connectivity and mechanical stiffness may be lower than those of Ca^2+^-cross-linked alginate hydrogels. One notable characteristic was that alginate gelation with Ln^3+^ occurred more abruptly and rapidly than with Ca^2+^, resulting in less homogeneous hydrogels. This difference may be attributed to the higher charge density of Ln^3+^ ions, which reduces their mobility in the alginate solution and promotes the formation of domains with varying cross-linking densities.^55^ On the other hand, when precoordinated lanthanide complexes were used as cross-linkers (Alg-Ln), the *G’* increased significantly. For example, the Alg-Eu hydrogel (Ln complex = 1 wt %) exhibited a *G’* of ∼ 24 kPa, more than an order of magnitude higher than that of Alg-CaN hydrogels. This result indicated that the enhanced mechanical properties arose from electrostatic interactions and complex-assisted cross-linking between lanthanide complexes and alginate chains, forming a more robust network structure.

The thermal stability of the Alg-Ln hydrogels was evaluated using differential scanning calorimetry (DSC). A slight increase in the melting temperature (*T*_m_) was observed for the Alg–Sm hydrogel (133.5 ± 6.2 °C) compared to Alg–Tb (118.3 ± 8.9 °C) and Alg–Eu (119.8 ± 7.5 °C) hydrogels (Figure 4c and Figure S24). However, the differences are relatively small and partially overlap within experimental error, indicating comparable thermal transitions among the Alg–Ln hydrogels. In addition to assessing thermal stability, the structural integrity of the hydrogels was assessed by immersion in an aqueous solution. The results showed that the Alg-Ln hydrogels retained over 20% of their structural integrity for 12 days (Figure S25). After 12 days of immersion, the Alg-Sm hydrogels showed a slower degradation rate than the Alg-Eu and Alg-Tb hydrogels. Specifically, the Alg-Sm hydrogel retained 45.5 ± 2.3% of its structural integrity compared to the Alg-Tb (22.5 ± 1.5%) and Alg-Eu (30.2 ± 2.6%) hydrogels (Figure 4d). The swelling behavior of the Alg-Ln hydrogels was further investigated by immersing the lyophilized hydrogels in water at 37 °C. All hydrogels reached equilibrium swelling within 2 h after immersion. Remarkably, the Alg-Tb hydrogel showed the highest swelling ratio (∼12) and water content (93.0 ± 5.9%) after 2 h, while the Alg-Sm hydrogel showed the lowest swelling ratio (∼8.3) and water content (81.0 ± 7.6%) (Figure 4e,f). To further evaluate the mechanical stability of hydrogels after rehydration, rheological measurements were performed and compared with those of the native hydrogels. The results showed that the *G’* values of the hydrogels before and after immersion were very similar (Figure S26 and Table S12). For example, the *G’* of Alg-Eu decreased slightly from 8820 ± 664 Pa to 8282 ± 452 Pa, while Alg-Tb and Alg-Sm hydrogels showed similarly small changes. Therefore, the hydrogel network structure was largely preserved after lyophilization and rehydration.

We also measured the luminescence spectra and PLQY of Alg-Ln hydrogels containing 0.75 wt % Ln complex after 2 h of rehydration in water (Figure S27). The results showed that the characteristic emission peaks of the Alg-Tb, Alg-Eu, and Alg-Sm rehydrated hydrogels were well preserved after immersion, and the PLQY values changed slightly compared to the pristine hydrogels (Table S7). These observations indicate that the hydrogels retained their luminescent properties after 2 h of immersion in water, further supporting the stability of the hydrogel network under aqueous conditions.

Both DSC and immersion tests indicated that Alg-Sm hydrogels exhibited a modest tendency toward improved thermal stability and slower degradation, which could be attributed to their highly cross-linked networks and more rigid structures. Additionally, the immersion results reflected the intricate interplay between pore size and swelling behavior of the hydrogels, with Alg-Sm hydrogel having the smallest pore size to resist water infiltration and maintain structural integrity during immersion.

Overall, three types of lanthanide complexes were used to cross-link alginate through electrostatic interactions, fine-tuning the structures and properties of the resulting hydrogels. The role of different lanthanide complexes in modulating the network, gelation time, pore sizes, cross-linking density, mechanical strength, thermal stability, and swelling capability of Alg-Ln hydrogels was illustrated quantitatively in the radial diagrams (Scheme 2). Among the three hydrogels, Alg-Sm hydrogels exhibited faster gelation time, smaller pore size, better mechanical strength, higher thermal stability, and lower swelling ratio compared to Alg-Tb and Alg-Eu hydrogels. The superior performance of the Alg-Sm hydrogels is likely attributed to the larger volume of the Sm complex, which increases the spacing between alginate polymers to facilitate the formation of additional electrostatic cross-linking points. In contrast, the Tb complex, which is the smallest of the three complexes, leads to lower *G’* values and correspondingly poorer mechanical properties. It should be noted that the size differences among the lanthanide ions are subtle, even when considering the packing arrangements of the molecules (Figure S28). Notably, these results demonstrate that even such minor variations can significantly influence the characteristics of hydrogel networks. According to the literature, parameters such as the p*K*_a_ of the metal aquo complex [Ln­(H_2_O)_n_]^3+^ and/or the ionic radius of Ln^3+^ indicate that lanthanide ions can tune key properties, including the Lewis acidity and reduction potential of the entire cluster.^56^ Therefore, synthesizing a complete series of lanthanide analogs for the hydrogel system will be essential for further investigation.

**Scheme 2:**
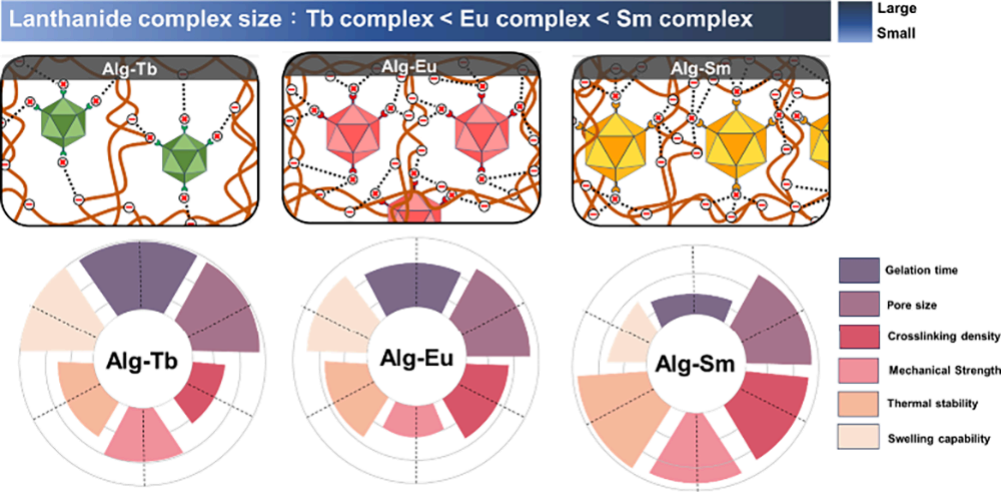
Schematic Illustrations of the Influence of Ln Complexes on the Networks and Properties of Alg-Ln Hydrogels

The electrostatic interactions between the Ln complexes and the alginate network provided the self-healing, shear-thinning, and injectable properties of the Alg-Ln hydrogels. The self-healing behavior of the Alg–Ln hydrogels was demonstrated by cyclic step-strain oscillatory shear measurements, alternating between 1% and 500% strain (Figure 5a). At high strain, the *G’’* exceeded the *G’*, indicating a temporary disruption of the polymer network. However, when the strain was reduced back to 1%, both *G’* and *G’’* quickly recovered to their original values, illustrating the inherent self-healing ability of Alg-Ln hydrogels. These results suggest that the electrostatic interactions within the network are robust enough to allow structural reforming following deformation, highlighting the potential of Alg-Ln hydrogels for applications requiring durable and resilient materials. An optical microscope image was further used to directly observe the self-healing process, showing that the interface between the cut sections decreased with increasing healing time within 30 min, and the interface was no longer visible after 60 min (Figure 5b). A compression test revealed that the recovery efficiency of the Alg-Ln hydrogels was time-dependent (Figure 5c and Figure S29). For instance, the recovery efficiency of the Alg-Eu hydrogel was 8.7%, 17.4%, 38.0%, 54.4%, and 84.8% recovery after 2, 4, 6, 8, and 10 h, respectively (Figure 5c). It was also noticed that Alg-Sm hydrogels presented a slower recovery time compared to Alg-Eu and Alg-Tb hydrogels, suggesting enhanced mechanical strength of the cross-linked network may impede self-repair mechanisms.

**Figure 5:**
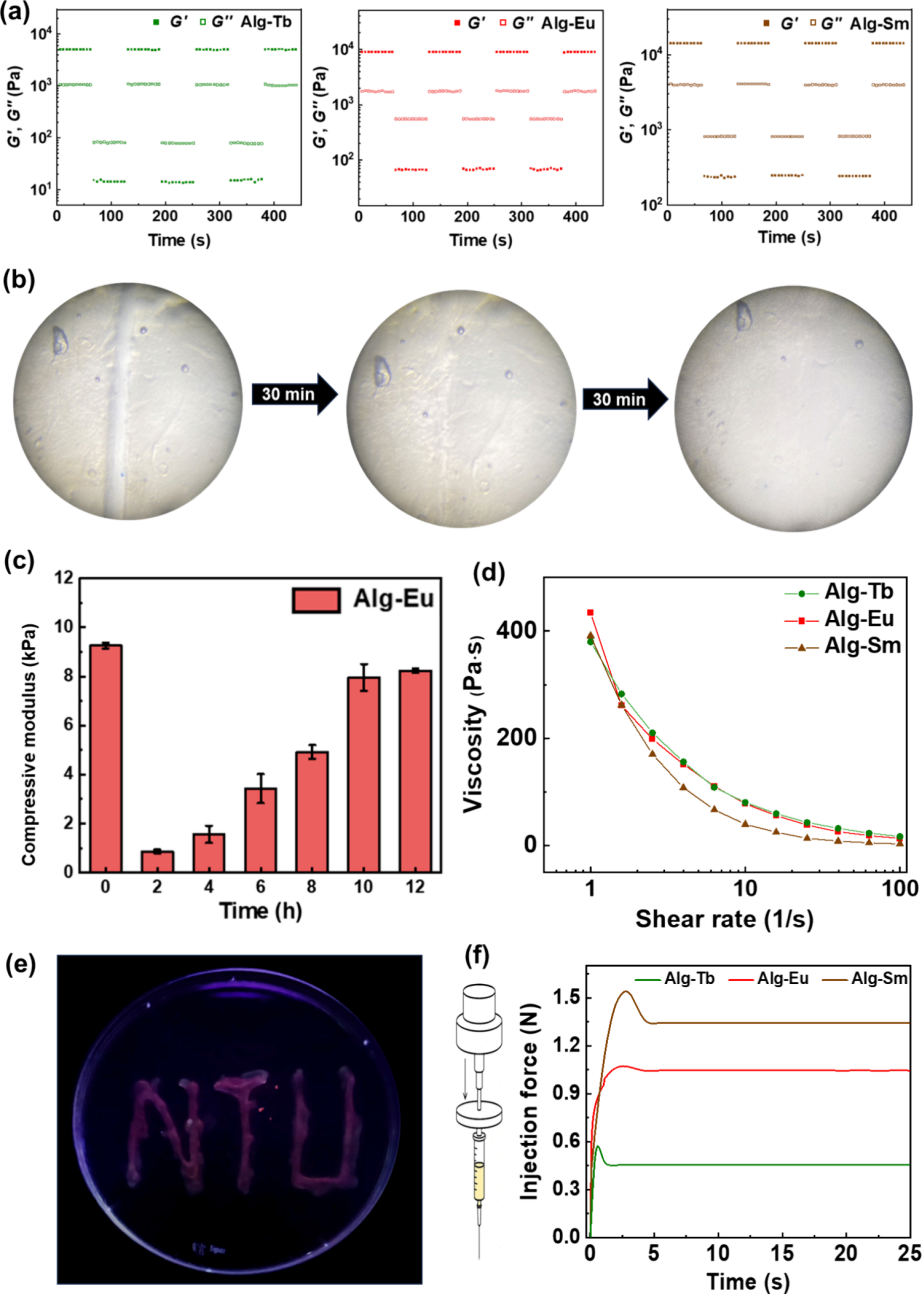
Self-healing, shear-thinning, and injectable behaviors of Alg-Ln hydrogels (with 0.75 wt % Ln complex). (a) *G’* and *G’’* of Alg-Ln hydrogels were recorded under the cyclic strain time sweep changes between strains of 1% and 500%. (b) Optical microscope images of the self-healing process at 25 °C. (c) The compressive modulus of hydrogel before and after healing for different time periods. (d) Continuous flow sweeps and stress-shear rate curves of Alg-Ln hydrogels. (e) Demonstrations of the Alg-Eu hydrogel with writing ability. (f) Schematic illustration of the setup of the injection force measurement and injection force profiles of Alg-Ln hydrogels.

The Alg-Ln hydrogels exhibited shear-thinning behavior as their viscosity decreased with increasing shear rate (Figure 5d). Taking advantage of the shear-thinning behavior, the injectability of the Alg-Eu hydrogel was demonstrated by extruding it through a syringe to form letters ″NTU″ (abbreviation of National Taiwan University), where the resulting hydrogel constructs exhibited pronounced red luminescence upon UV irradiation at 365 nm (Figure 5e). To quantify the injectability of Alg-Ln hydrogels, the force required for extrusion was measured by filling the hydrogels into a syringe with an 18G needle and extruding at a flow rate of 1 mL/min (Figure 5f). The injection forces for the hydrogels Alg-Tb, Alg-Eu, and Alg-Sm hydrogels were about 0.48, 1.03, and 1.33 N, respectively. These results correlated with the microstructure and mechanical properties of the hydrogels, suggesting that higher extrusion forces were required for the Alg-Sm hydrogel due to its denser and more robust network. In summary, the analyses of self-healing and shear-thinning properties of the Alg-Ln hydrogels highlighted their potential for use as injectable luminescent materials for 3D printing and additive manufacturing, where tunable mechanical properties and structural integrity are critical. The observed relationships between microstructure, mechanical strength, and injectability provide valuable insights for optimizing these hydrogels for specific application requirements.

Compared to the reported covalently cross-linked PAM/DMC-Ln­(DPA)_3_ ^28^ and poor luminescent PG/Ln­(PA)_3_ hydrogels,^29^ our study advances the application of precoordinated lanthanide complexes in hydrogel formation by introducing positively charged luminescent Ln complexes with lanthanides of differing ionic radii. This design facilitates the electrostatic cross-linking of negatively charged alginate polymers, enabling the formation of mechanically stable hydrogels. These precoordinated lanthanide complexes serve as dual-functional cross-linkers, acting both as luminophores and dynamic cross-linkers to construct versatile structures of alginate hydrogels. The luminescent properties of the Ln complexes are preserved within the inherently water-rich environment of hydrogels. As chemical cross-linkers, the Ln complexes construct a cross-linked network within the hydrogel matrix spontaneously through electrostatic interactions, allowing the hydrogels to be self-healing and injectable. Furthermore, the strategic selection of lanthanide ions with differing ionic radii enables tunable luminescence, microstructures, mechanical properties, and swelling behavior of the hydrogels, broadening their applicability across diverse domains. Taken together, careful design and incorporation of lanthanide complexes as multifunctional cross-linking agents can effectively customize the physicochemical properties and performance of hydrogels. These self-healable and injectable lanthanide-containing hydrogels offer significant potential for advanced biomedical applications (e.g., sensing and imaging) by being integrated into cutting-edge biomedical devices for real-world applications.

### 3.4 The Alg-Ln Lyophilized Hydrogels for Metal Ion Sensing

Lanthanide-containing luminescent hydrogels have been widely used for sensing metal ions due to their low cost, high sensitivity, and ease of use.^57,58^ Here, Alg-Eu lyophilized hydrogel was used as a representative luminescent sensor for metal ions due to its superior PLQY compared to the others.

To clarify the practical implications of using lyophilized hydrogels for sensing, the Alg–Eu hydrogels in this study were primarily designed as disposable, single-use sensors operating in the dried state. Lyophilization suppresses O–H vibrational quenching of Eu^3+^ emission, enhances luminescence intensity, and creates a highly porous structure that enables rapid rehydration and efficient ion diffusion during detection. Although reuse is technically feasible due to the reversible electrostatic cross-linking network, repeated sensing cycles may cause partial loss of Eu^3+^ complexes through competitive coordination with Cu^2+^ ions, resulting in reduced signal reliability. Therefore, single-use operation provides the most stable and reproducible sensing.

Alg-Eu lyophilized hydrogels were immersed in solutions containing different metal ions (i.e., Na^+^, K^+^, Cu^2+^, Ca^2+^, Co^2+^, Ni^2+^, Mg^2+^, Zn^2+^, Fe^2+^, Fe^3+^, and Al^3+^) (10^–3^ M) for 1 h. After incubation, the samples were lyophilized before recording the photoluminescence spectra (Figure 6a). The changes in the luminescence intensity of the lyophilized hydrogels upon interaction with these metal ions were analyzed, and the results were visualized under UV irradiation. Among the metal ions tested, Cu^2+^ led to a significant reduction in the luminescence intensity (∼ 75% at peak of 616 nm) of the Alg-Eu lyophilized hydrogels (Figure 6b). These luminescence data were further processed using linear discriminant analysis (LDA) to assess the ability of the Alg-Eu lyophilized hydrogel to differentiate between metal ions. The results showed that Cu^2+^ ions caused a notable quenching of luminescence in the presence of lyophilized Alg-Eu hydrogel, distinguishing them from the other tested metal ions through LDA (Figure 6c).

**Figure 6:**
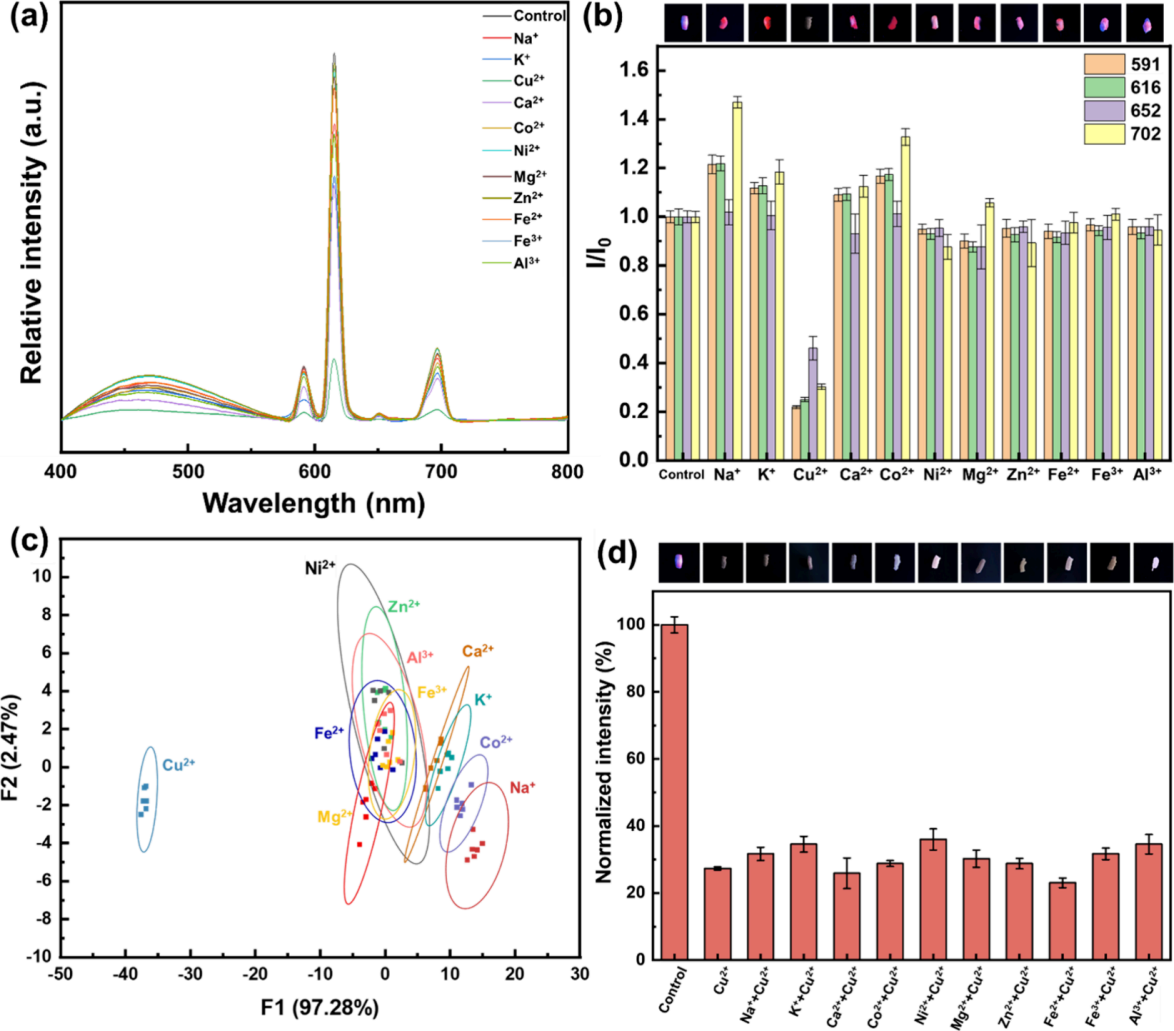
(a) Luminescence spectra of the Alg-Eu lyophilized hydrogels after immersing in different metal ion solutions. (λ_ex_= 360 nm) (b) Photos and luminescence responses of the Alg-Eu lyophilized hydrogels after immersing in different metal ion solutions. (c) Score plot for different metal ion sensing of Alg-Eu lyophilized hydrogels obtained from LDA (*n* = 6). (d) Photos and luminescence responses of the Alg-Eu lyophilized hydrogels after immersing in Cu^2+^ ion solution in the presence of other metal ions. Normalized the peak intensity at 616 nm. The photos were taken under UV irradiation at 365 nm.

The luminescence quenching phenomenon observed in Alg-Ln hydrogels upon exposure to Cu^2+^ ions can be attributed to the competition effect.^59,60^ The phenomenon of luminescence quenching observed in Alg-Ln hydrogels upon exposure to Cu^2+^ ions can be explained through energy transfer interference mechanisms similar to those previously reported for other lanthanide-based systems.^61,62^ Initially, the excitation energy is absorbed by the chelating ligands, which subsequently transfer this energy to the central Eu^3+^ ions, resulting in the characteristic red emission. Upon immersion in solutions containing Cu^2+^ ions, Cu^2+^ ions have a high affinity toward carboxylate groups,^63^ forming competitive coordination complexes that compromise the structural integrity of the original Eu^3+^ coordination sphere. Consequently, the effective energy transfer from the ligand to Eu^3+^ ions is compromised, resulting in a notable decrease in luminescence intensity, which enables the selective sensing of Cu^2+^ ions in the Alg-Ln hydrogel systems.

To directly test whether Cu^2+^ ions can displace Eu^3+^ ions from Alg-Eu coordination sites, the immersion leaching experiment was performed. Specifically, Alg-Eu hydrogels (100 μL) were soaked in 1 mL aqueous solutions of copper­(II) chloride (CuCl_2_), sodium chloride (NaCl), or water for 1 h at room temperature. The Eu^3+^ content in the supernatants was quantified by inductively coupled plasma mass spectrometry (ICP-MS) using an external calibration. The CuCl_2_ solution contained 27.2 ppm Eu^3+^ ions, while the NaCl solution had only 0.1 ppm, and water remained below the detection limit. These results clearly show that Cu^2+^ ions selectively promotes the release of Eu^3+^ ions from the hydrogel, in contrast to the Na^+^ ions and water controls, supporting the proposed mechanism of Eu^3+^ coordination competition with Cu^2+^ ions.

To further evaluate the practical applicability of the Alg-Eu lyophilized hydrogel as a selective sensing platform for Cu^2+^ ions, a competitive detection test was performed in the presence of various coexisting metal ions. Specifically, mixtures with Cu^2+^ ions and other selected metal ions were prepared, and the resulting luminescence intensities of the Alg-Eu lyophilized hydrogels were evaluated. As shown in Figure 6d, the luminescence intensity consistently decreased upon the addition of Cu^2+^ ions, regardless of the presence of the other metal ions. This result demonstrates that the luminescence quenching phenomenon triggered by Cu^2+^ ions was not affected by the simultaneous presence of commonly occurring interfering metal ions, confirming the reliability and specificity of the Alg-Eu lyophilized hydrogel-based sensor system for Cu^2+^ detection. These results underline the potential of Alg-Eu lyophilized hydrogels as selective and efficient sensors for Cu^2+^ ions in environmental or analytical applications.

The limit of detection (LOD) of the Alg-Eu lyophilized hydrogel in sensing Cu^2+^ was further determined, where the Alg-Eu lyophilized hydrogel was immersed in Cu^2+^ solutions with varying concentrations (Figure S30).^64,65^

The LOD of the Alg-Eu lyophilized hydrogel was calculated by the following eqs 2 and 3:

$$LOD=\frac{3\sigma }{K_{sv}}$$ 2

$$\sigma =\frac{F_{SE}}{F_{0}}$$ 3

Where F_SE_ was the standard error of the fluorescence intensity of the blank sample, and F_0_ was the luminescence intensity of Alg-Eu lyophilized hydrogel. The quenching constant K_SV_ was calculated according to the following eq 4:

I0I=KSV[M]+1 4

Where I_0_/I is the measurement of luminescence intensity before and after sensing, [M] is the molar concentration of Cu^2+^, and K_SV_ is the quenching constant. After the examination, the quenching constant K_SV_ was calculated to be 5.7 × 10^4^ L/mol, and the LOD of Cu^2+^ for Alg-Eu lyophilized hydrogel was 2.9 × 10^–7^ M.

To provide direct evidence for coordination changes after Cu^2+^ treatment, XPS analysis was performed on the lyophilized hydrogel before and after immersion in Cu^2+^ solution (Figure S31). As shown in the O 1s spectra, clear differences were observed after Cu^2+^ treatment. The O–Eu contribution decreased significantly, while a new O–Cu component appeared and became dominant. Quantitative fitting showed that the O–Eu peak area (1539) was much smaller than that of O–Cu (32566). These results indicated that Cu^2+^ effectively competed with Eu^3+^ for coordination with oxygen-containing groups, leading to a reorganization of the coordination environment.

Several lanthanide-containing hydrogels have been reported for the selective detection of Cu^2+^ ions (Table 1). Among the records, the Eu­(DPA)_3_@Lap-Tris platform showed an exceptionally low LOD of 92 nM for Cu^2+^ ions, which is primarily due to the fact that the coordination ligand significantly increases the luminescence intensity and thus enables a lower detection limit.^66^ Here, this Alg-Eu lyophilized hydrogel sensing system presented a comparable LOD of 290 nM, representing either superior or equivalent performance compared to the established lanthanide-containing hydrogel systems in Table 1. Regarding sensitivity, the Alg-Eu lyophilized hydrogel has shown a remarkable fluorescence quenching constant (K_SV_= 5.7 × 10^4^ L/mol), outperforming some of the previously reported systems, highlighting its superior responsiveness to low Cu^2+^ concentrations. In addition, selectivity analysis shows that this Alg-Eu lyophilized hydrogel has significant specificity toward Cu^2+^ ions. Its luminescence quenching effectiveness remains remarkably stable even in the presence of potentially interfering metal ions, including Fe^3+^, Na^+^, K^+^, Mg^2+^, and Ca^2+^, showing its practical applicability under complex environmental conditions. Overall, the low LOD (290 nM), high K_SV_ (5.7 × 10^4^ L/mol), and robust selectivity of Alg-Eu lyophilized hydrogel have significant specificity toward Cu^2+^ sensing, making it a suitable candidate for Cu^2+^ trace monitoring in the environment and industry.

**Table 1:** Lanthanide-Containing Hydrogels for Cu^2+^ Ion Detection

sensing elements	polymeric matrix	K_sv_(L/mol)	detection ranges (M)	LOD	ref.
Eu(DPA)_3_@Lap-Tris^a^	polyvinyl alcohol		10^–6^∼10^–5^	9.2 × 10^–8^ M	^66^
Eu(DPA)_3_·2H_2_O andTb(DPA)_3_·2H_2_O^b^	cellulose		10^–7^∼10^–6^		^74^
EuL^c^	polyvinyl alcohol, polyacrylamide-copolyacrylic acid		10^–6^∼10^–4^	3.6 × 10^–6^ M	^75^
T-Ln/U-Ln^d^				10^–2^ M	^76^
ME-IPA@SA-TbZn^e^	SA^f^		10^–3^∼0	1.3 × 10^–6^ M	^77^
Ln^3+^-ligand (Ln = Eu and Tb)^g^	poly(acrylamide), poly(methacrylic acid) and MBAA^h^			10^–1^ M	^78^
Tb^3+^@Lap^i^	PG/PDA^j^	5.3 × 10^4^	10^–6^∼10^–5^	9.2 × 10^–5^ M	^58^
Eu complex	alginate	5.7 × 10^4^	10^–6^∼10^–5^	2.9 × 10^–7^ M	this work

a DPA= dipicolinic acid (or pyridine-2, 6-dicarboxylic acid).

b DPA= dipicolinic acid (or pyridine-2, 6-dicarboxylic acid).

c L= 5,5′-(ethane-1,2-diylbis­(oxy)) diisophthalic acid.

d *T* = thymidine; G= uridine.

e ME= melamine; IPA= isophthalic acid; SA= sodium alginate.

f SA= sodium alginate.

g Ligand= terpyridine.

h MBAA= *N*,*N*’-Methylenebis­(acrylamide).

i Lap= laponite.

j PG= polyethylenimine-modified gelatin; PDA= polydextran aldehyde.

The Alg-Eu hydrogel can be readily fabricated into freestanding or lyophilized monolithic forms, allowing its direct use as a solid-state luminescent probe for on-site Cu^2+^ detection in aqueous environments. For example, it may be applied to rapid screening of Cu^2+^ contamination in drinking water sources,^67,68^ industrial wastewater discharge,^69^ agricultural irrigation systems,^70,71^ or aquaculture environments), where copper accumulation can pose ecological and health risks. Importantly, the low detection limit of the Alg-Eu hydrogel enables the identification of Cu^2+^ at trace-level concentrations in the micromolar range, which is relevant to environmental safety guidelines and biological systems where excess copper may induce oxidative stress and toxicity.^72,73^ Compared with solution-based molecular probes, the hydrogel platform provides a mechanically stable, easy-to-handle solid format with a visual luminescence readout under UV irradiation, facilitating portable, semiquantitative field analysis.

## 4 Conclusions

A series of luminescent alginate hydrogels was successfully fabricated using precoordinated lanthanide complexes as luminophores and dynamic cross-linkers. Enhanced structural integrity was achieved when using precoordinated lanthanide complexes, compared to employing Ln­(NO_3_)_3_ as an ionic cross-linker for alginate hydrogel formation. This study also revealed that the structures and properties of these Alg-Ln hydrogels could be precisely adjusted by varying the lanthanides in the complexes. Among the hydrogels, Alg-Sm demonstrated faster gelation time, a denser network, and increased mechanical strength compared to Alg-Tb and Alg-Eu hydrogels. This was attributed to the larger Sm complex that can increase the spacing between polymer chains and facilitate the formation of additional electrostatic cross-linking points. These Alg-Ln hydrogels also exhibited remarkable dynamic properties, including self-healing, shear thinning, and injectability. Furthermore, the luminescent Alg-Eu hydrogels were sensitive and selective luminescent sensors for Cu^2+^ ion detection, achieving a LOD of 2.9 × 10^–7^ M. Although the PLQY values of the Eu complex (2.22%) and Alg–Eu hydrogel (2.01%) remain relatively low due to water-induced quenching, future improvements may be achieved by employing ligands^79–81^ with stronger antenna effects or by introducing hydrophobic or sterically protected coordination environments to further shield Ln^3+^ ions from O–H vibrational quenching pathways. Such molecular engineering strategies could substantially improve the luminescence efficiency of next-generation Alg–Ln hydrogels.

Taken together, this study demonstrates the attractive strategy of using precoordinated lanthanide complexes to cross-link alginate, producing luminescent alginate hydrogels with customizable characteristics. The versatile structural and dynamic features of Alg-Ln hydrogels make them promising luminescent materials for diverse applications, such as imaging and sensing.

## Supplementary Material



## Notes

The Supporting Information contains additional characterization and structural data, including synthetic schemes and proposed reaction mechanisms, ^1^H NMR spectra, FTIR spectra, excitation and lifetime measurements, SEM images, zeta potential analyses, rheological behavior, DSC curves, and degradation studies. Single-crystal X-ray diffraction data for the Tb complex, SHAPE analysis, selected bond lengths and angles, pore structure analyses, mechanical properties, and calibration plots for Cu^2+^ sensing in Alg-Eu hydrogels are also provided (PDF)
