# Supplementary material for: Tailoring Alginate Hydrogels via Precoordinated Lanthanide Complexes as Dynamic Cross-Linkers
Source: Biomacromolecules. 2026 May 5;27(6):3594–611. doi: 10.1021/acs.biomac.5c02784 (PMC13250910; doi:10.1021/acs.biomac.5c02784)
Supplement: Supplementary file 1 [file bm5c02784_si_001.pdf]

Tailoring Alginate Hydrogels via Precoordinated Lanthanide Complexes as Dynamic Cross-Linkers

Yu-Chia Su[a], Li-Hsin Chang[b], Tai-Lin Wu[a], Po-Heng Lin[b]*, and Yi-Cheun Yeh [a]*

[a] Institute of Polymer Science and Engineering, National Taiwan University, Taipei 10617, Taiwan.

[b] Department of Chemistry, National Chung Hsing University, 250 Kuo Kuang Rd., Taichung 402, Taiwan

Corresponding authors: poheng@dragon.nchu.edu.tw; yicheun@ntu.edu.tw

(L^{SR}/L^{RR})

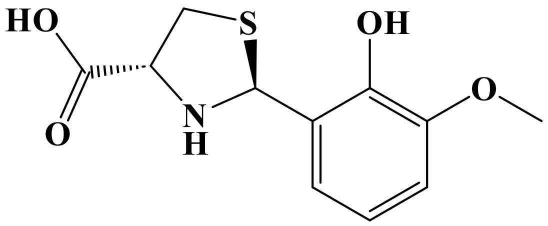

(2S,4R)-2-(2-hydroxy-3-methoxyphenyl)thiazolidine-4-carboxylic acid

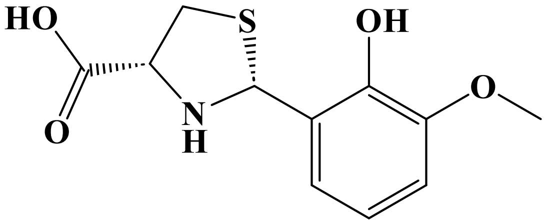

(2R,4R)-2-(2-hydroxy-3-methoxyphenyl)thiazolidine-4-carboxylic acid

(R-tac)

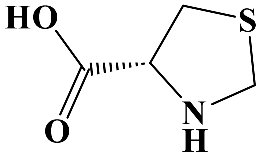

(R)-thiazolidine-4-carboxylic acid

$$L^{SR}/L^{RR} + LnCl_3 \bullet H_2O \xrightarrow{\text{EtOH/acetone}} [Ln_2(R\text{-}tac)_4(H_2O)_8] \bullet 6Cl$$

Ln = Tb, Eu or Sm   Ln = Tb, Eu or Sm

Scheme S1. Synthetic routes of complex **1-3**.

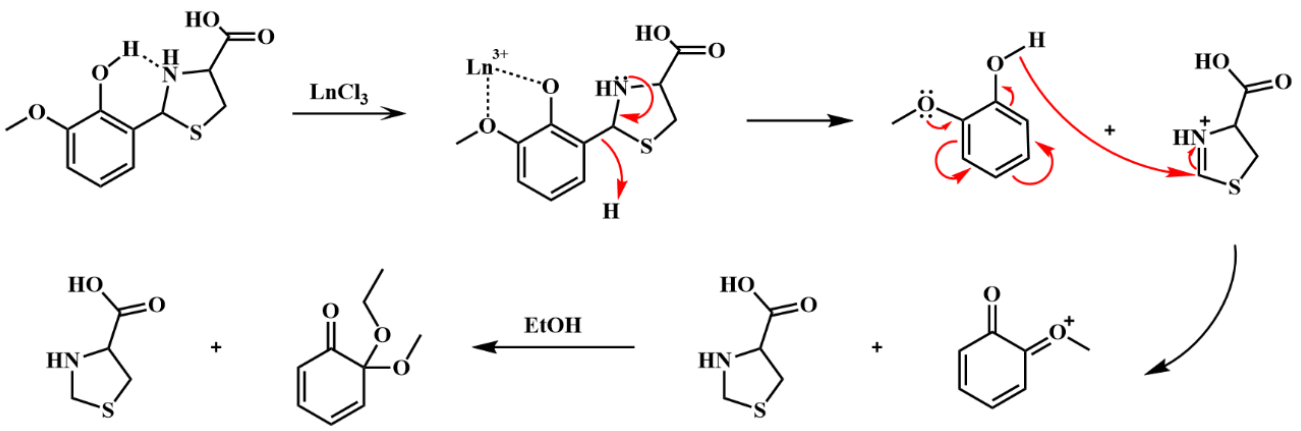

Scheme S2. Proposed mechanism for the synthesis of R-tac ligand.

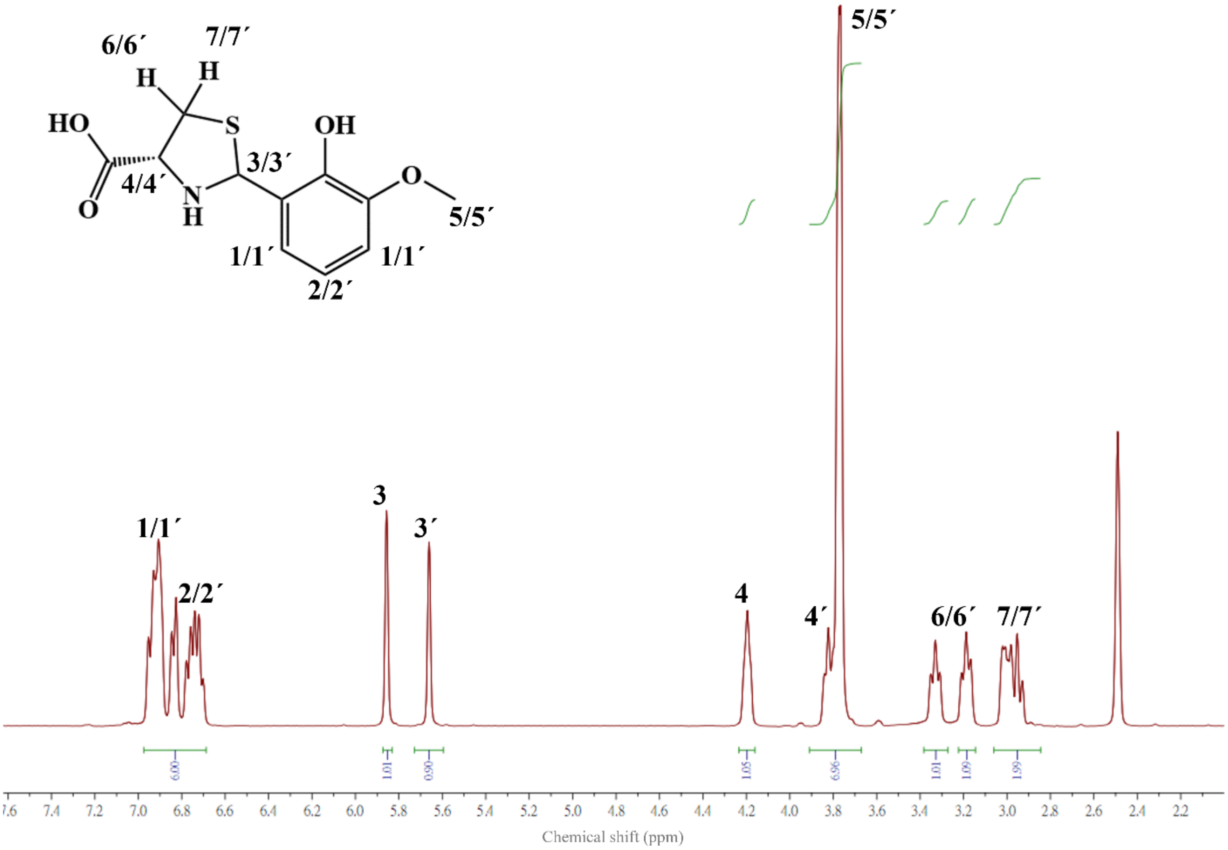

Figure S1. ^{1}H NMR spectrum of L^{SR}/L^{RR} in DMSO-d_6 solution. (400 MHz)

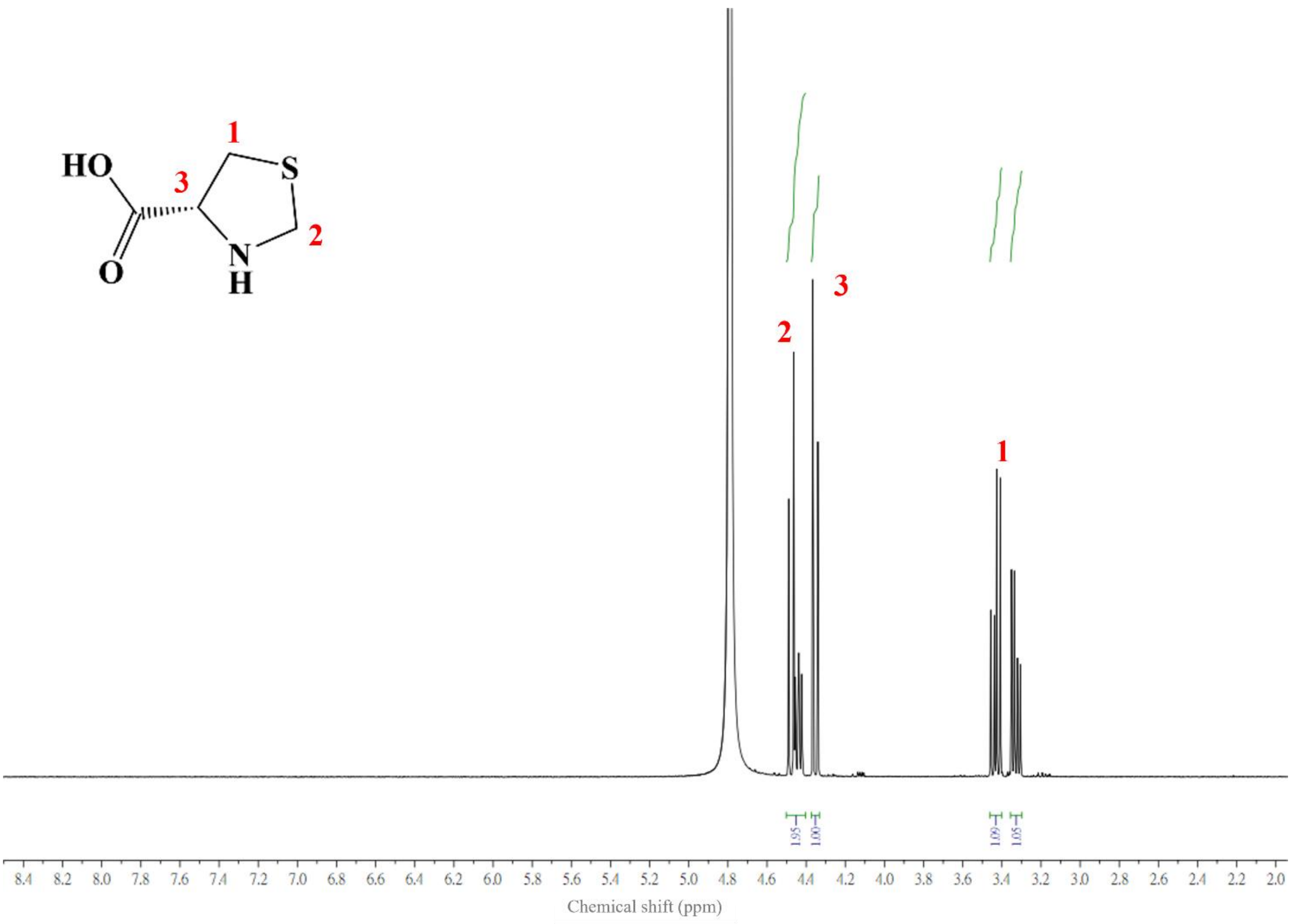

Figure S2. ^{1}H NMR spectrum of R-tac in D_2O solution. (400 MHz)

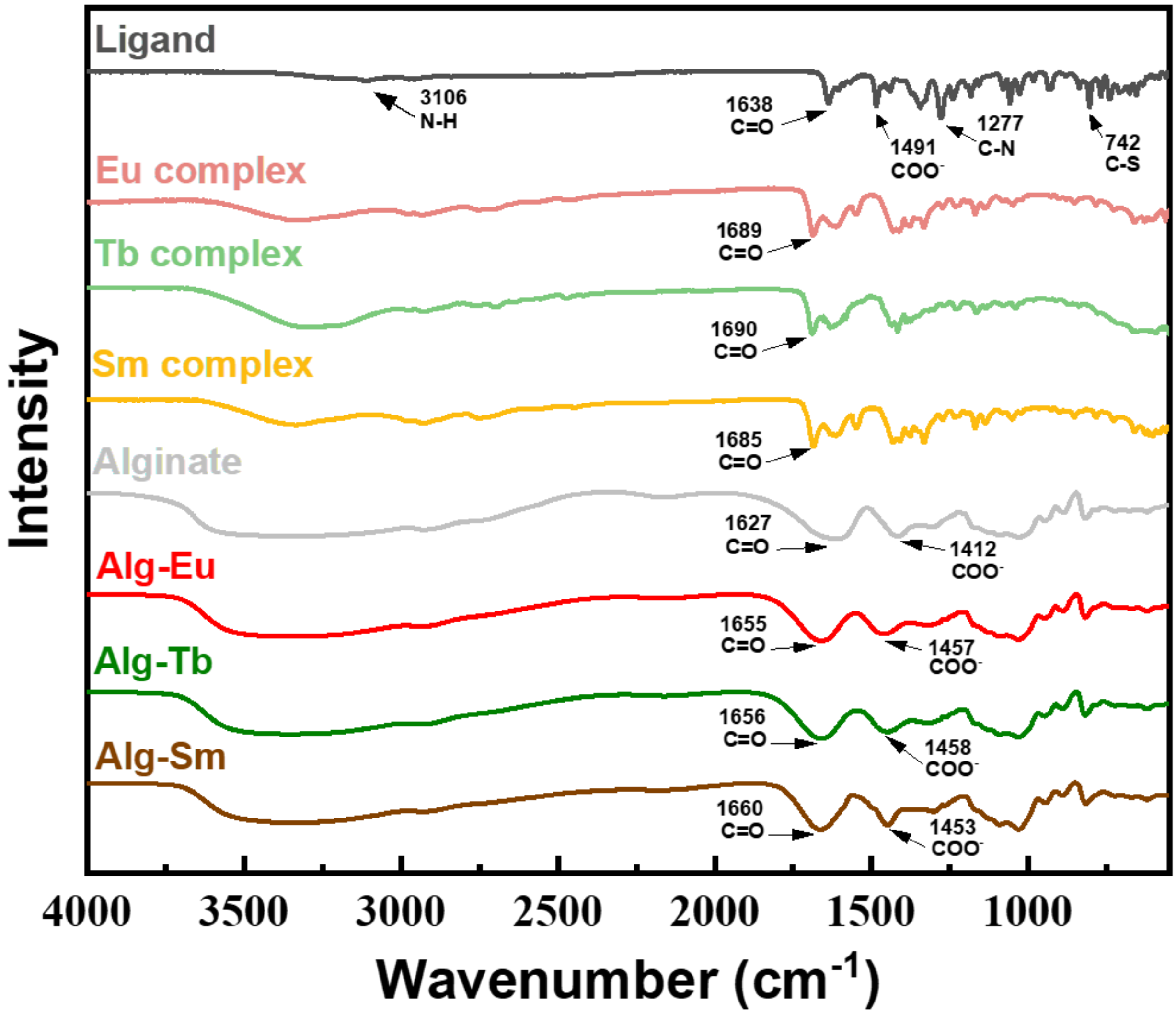

Figure S3. FTIR spectra of ligand, Ln complex, Alg, and Alg-Ln.

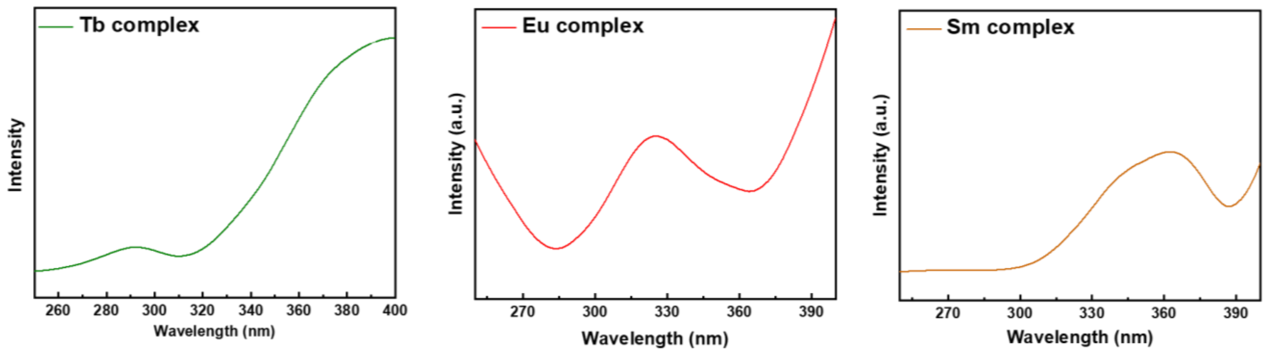

Figure S4. Excitation spectra of Tb, Eu, and Sm complexes monitored at λem = 543, 616, and 642 nm, respectively.

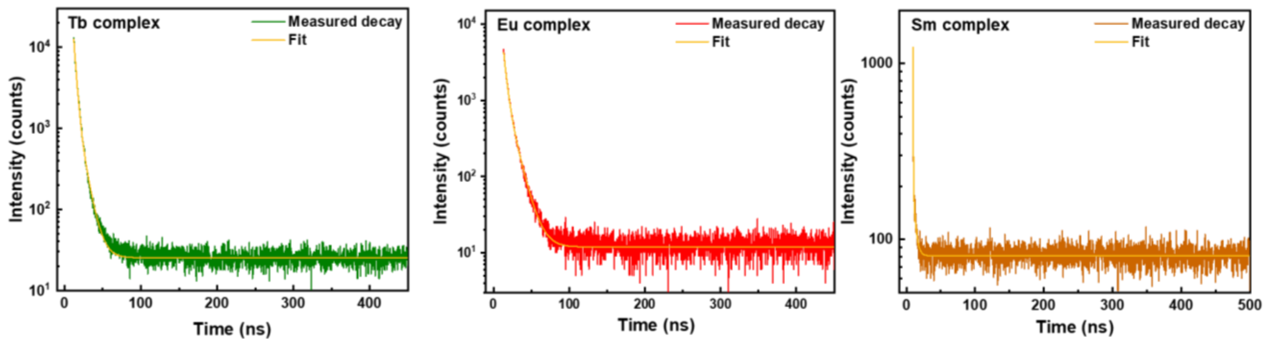

Figure S5. Life-resolved measurement of Ln complexes.

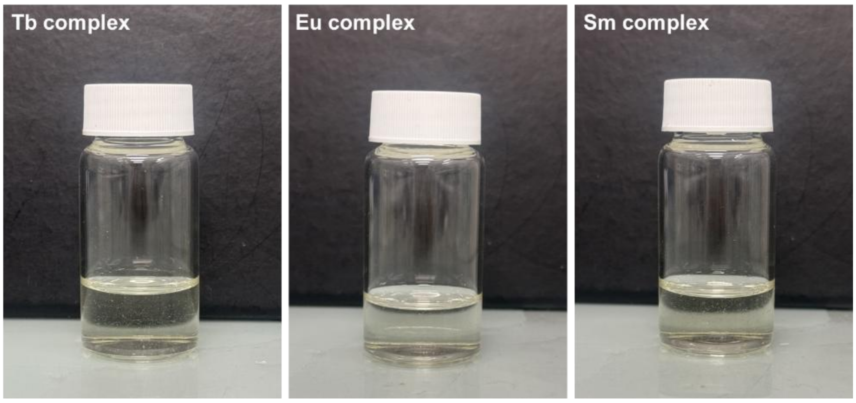

Figure S6. Dispersion of Ln complexes in water, where 50 mg of the complex was dispersed in 5 mL of water.

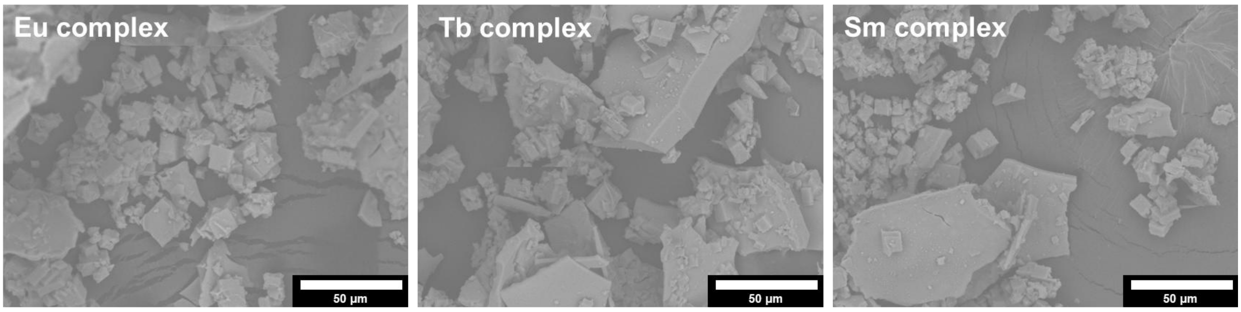

Figure S7. Representative SEM images of Ln complexes.

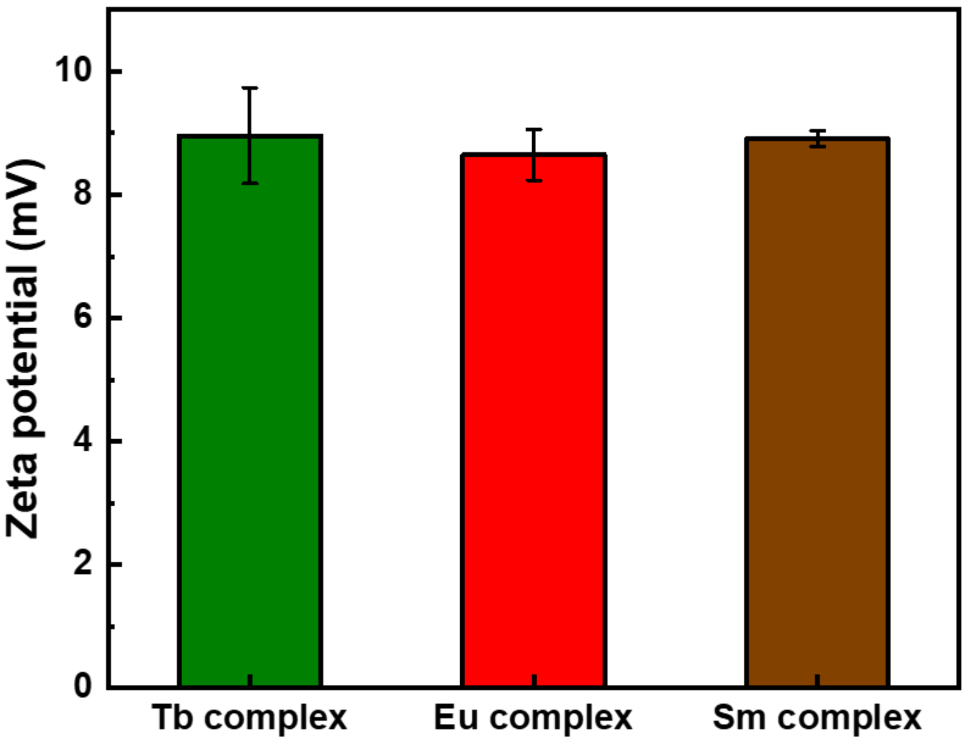

Figure S8. Zeta potentials of Ln complexes.

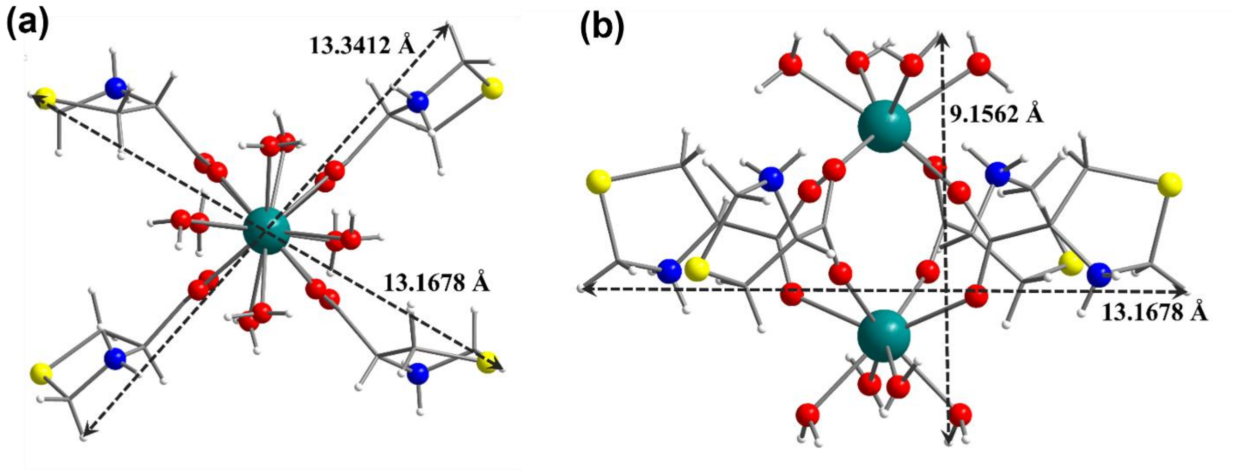

Figure S9. (a) Top and (b) side views of complex **1**. The dashed arrows indicate the molecular dimensions. Color code: dark green, Tb; red, O; blue, N; grey, C; yellow, S; white, H.

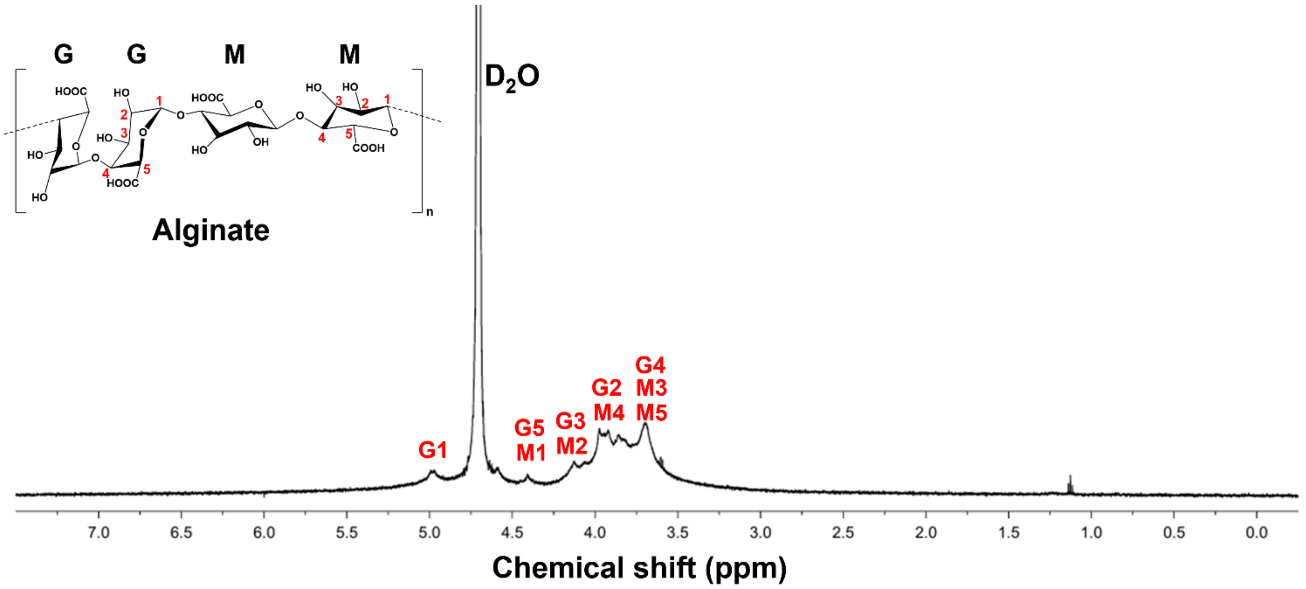

Figure S10. ^{1}H NMR spectrum of alginate. β-D-mannuronic acid (M) and α-L-guluronic acid (G).

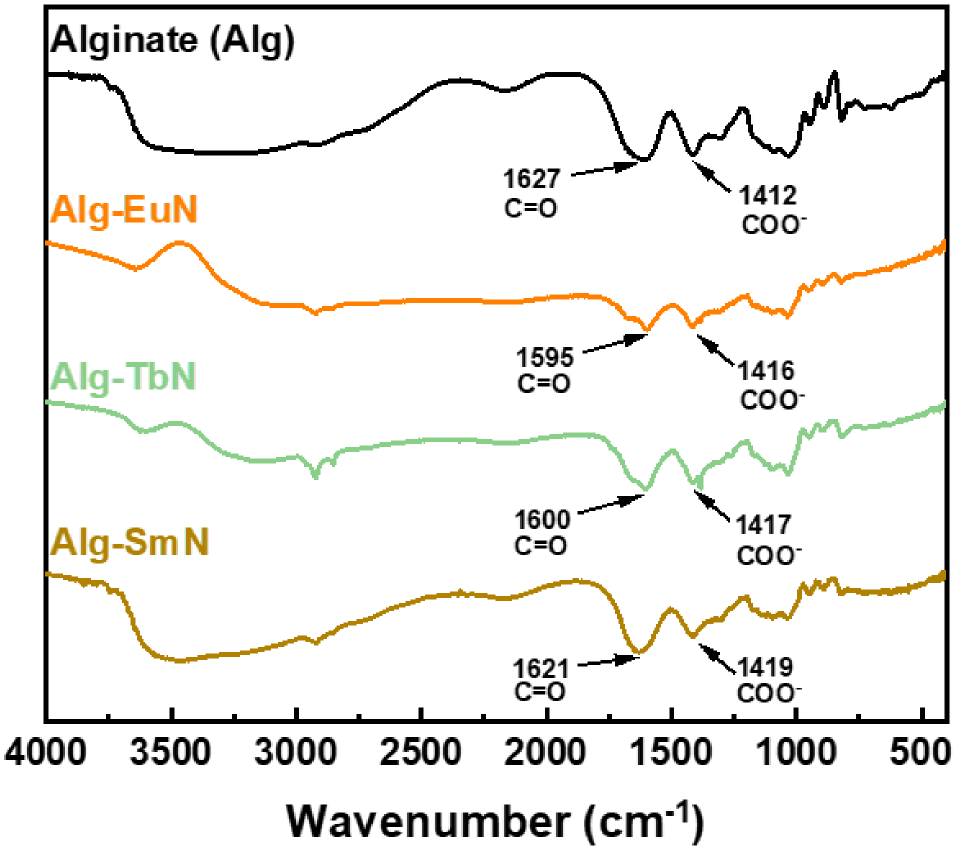

Figure S11. FTIR spectra of Alg and Alg-LnN.

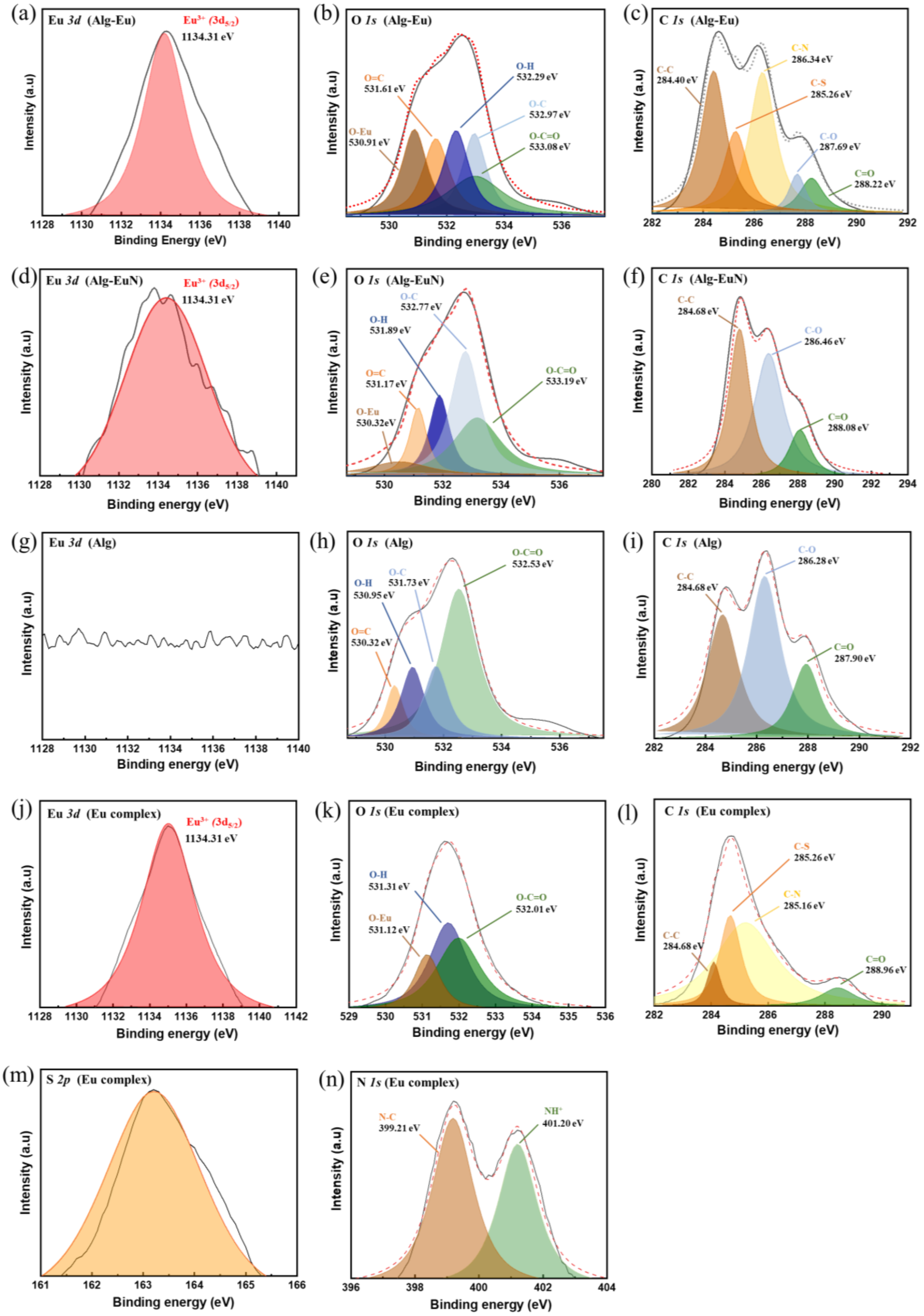

Figure S12. XPS spectra of (a–c) Alg–Eu, (d–f) Alg–EuN, (g–i) alginate (Alg), and (j–n) Eu complex.

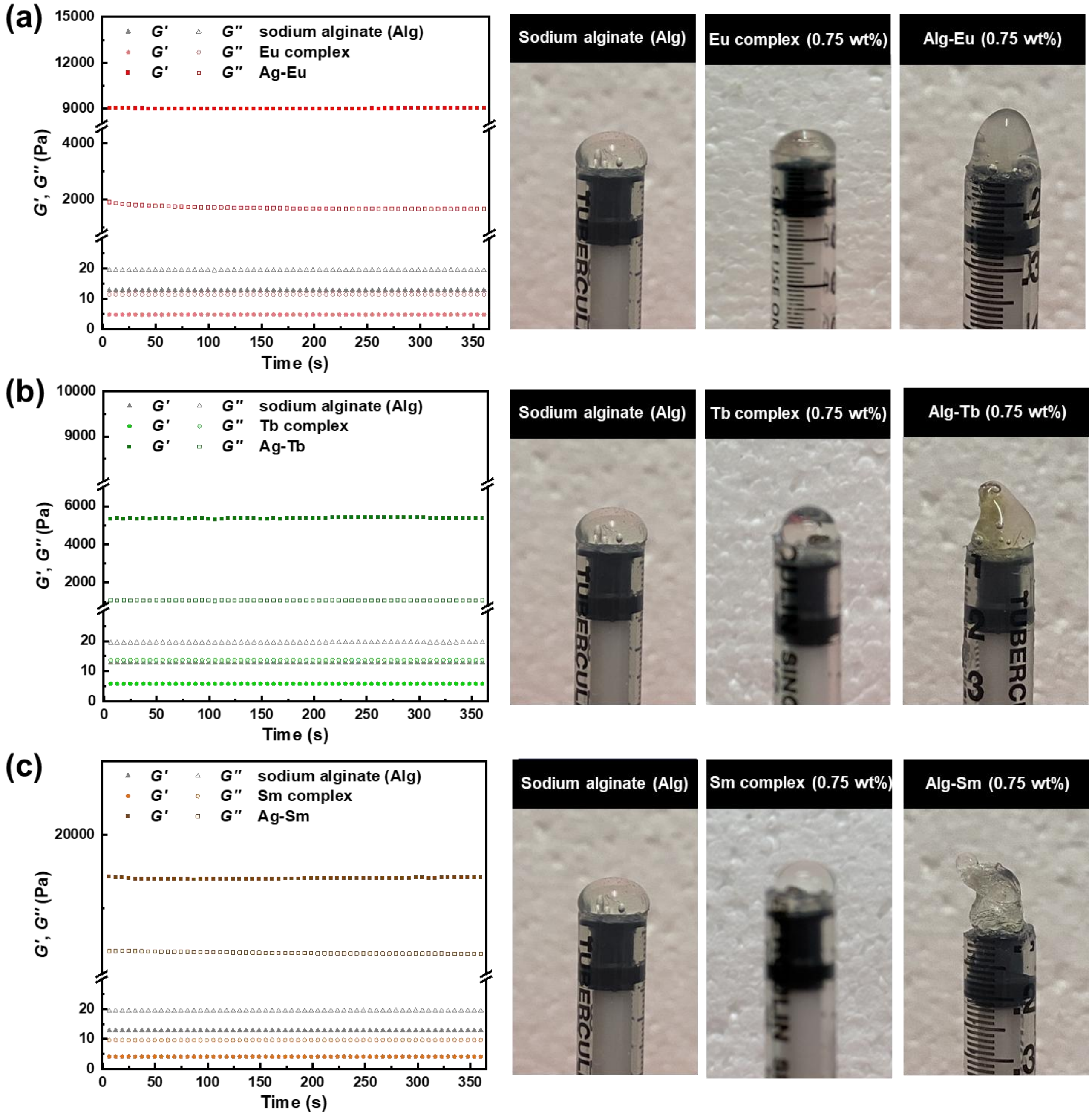

Figure S13. Photos and continuous time sweeps of Alg, Ln complex, and Alg-Ln samples.

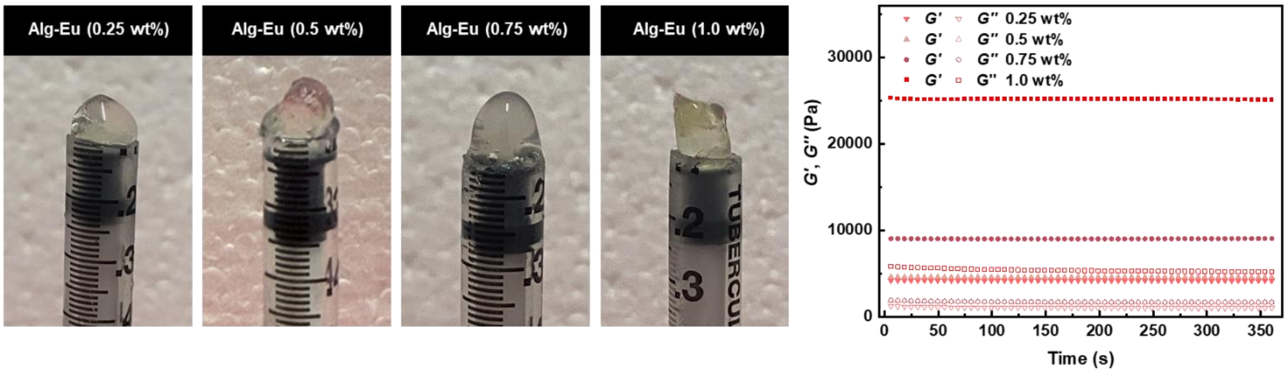

Figure S14. Continuous time sweeps and photos of Alg-Eu hydrogels prepared with different wt% of Eu complex.

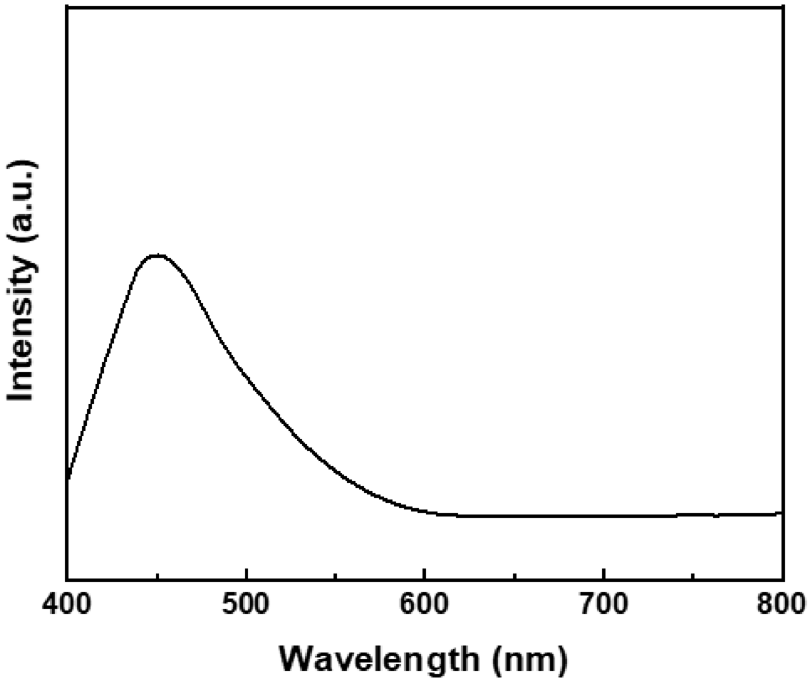

Figure S15. Luminescence spectrum of the alginate hydrogel, where alginate was crosslinked with Ca^{2+}.

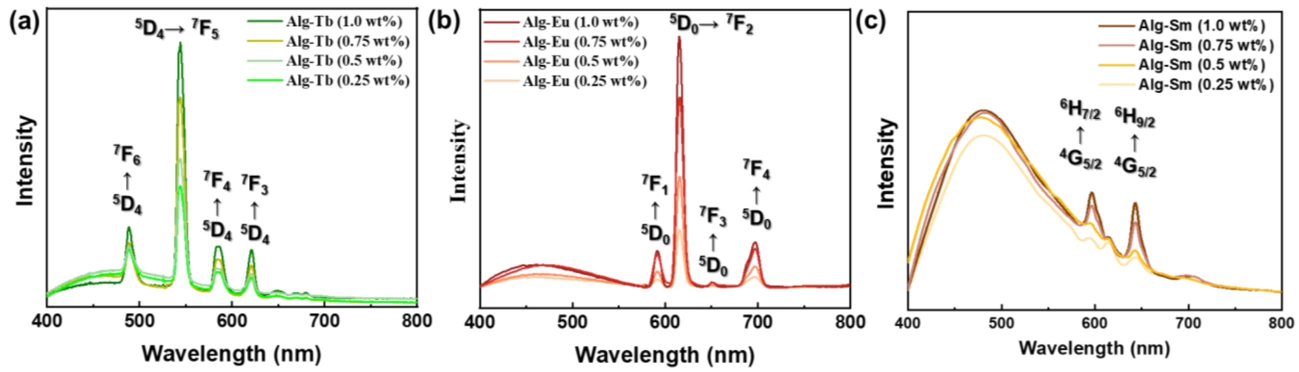

Figure S16. Luminescence spectra of (a) Alg-Tb, (b)Alg-Eu, and (c) Alg-Sm lyophilized hydrogels with different concentrations of Ln complexes. (λ_{ex} = 360 nm)

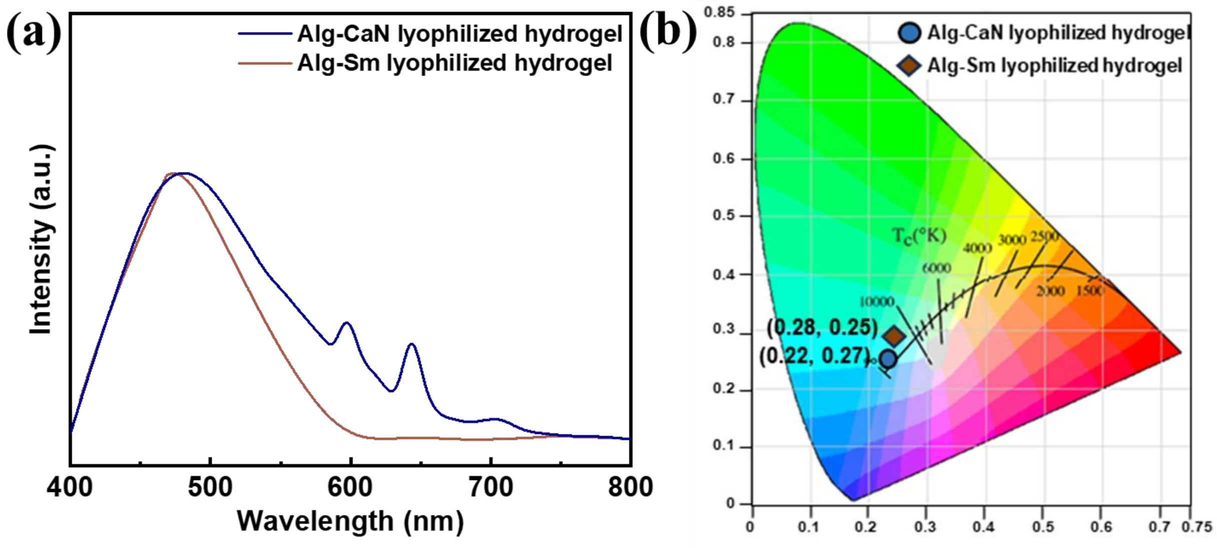

Figure S17. (a) Luminescence spectrum of the alginate hydrogel, where alginate was crosslinked with Ca^{2+} (b) CIE chromaticity coordinates of Alg-Sm, Alg-CaN. Alg-M hydrogels were prepared with alginate (5 wt%), and Ln complex or $CaSO_4$ (0.75 wt%).

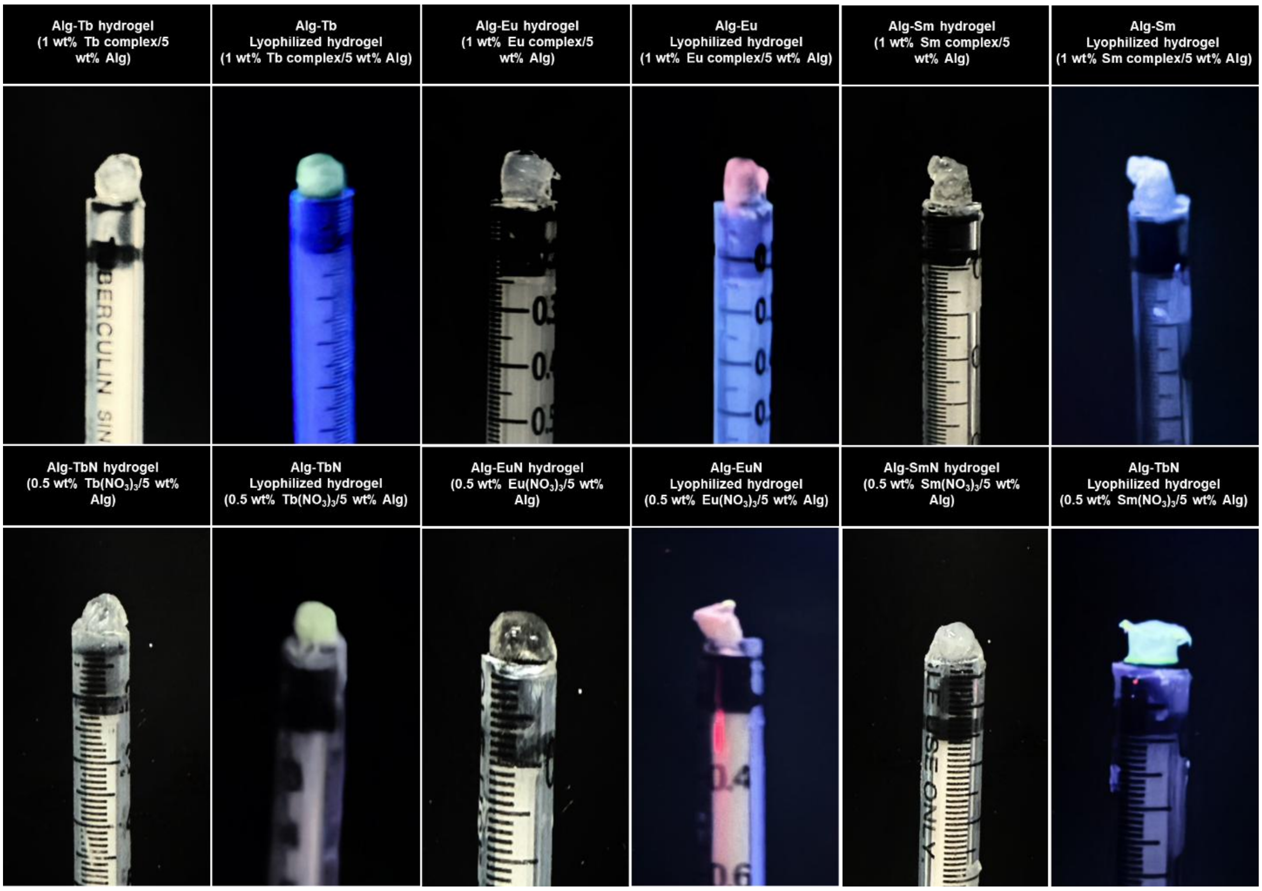

Figure S18. Photos of Alg-Ln and Alg-LnN samples, with the same moles of Ln ions in the samples. Alg-LnN samples were alginate crosslinked with $Ln(NO_3)_3$.

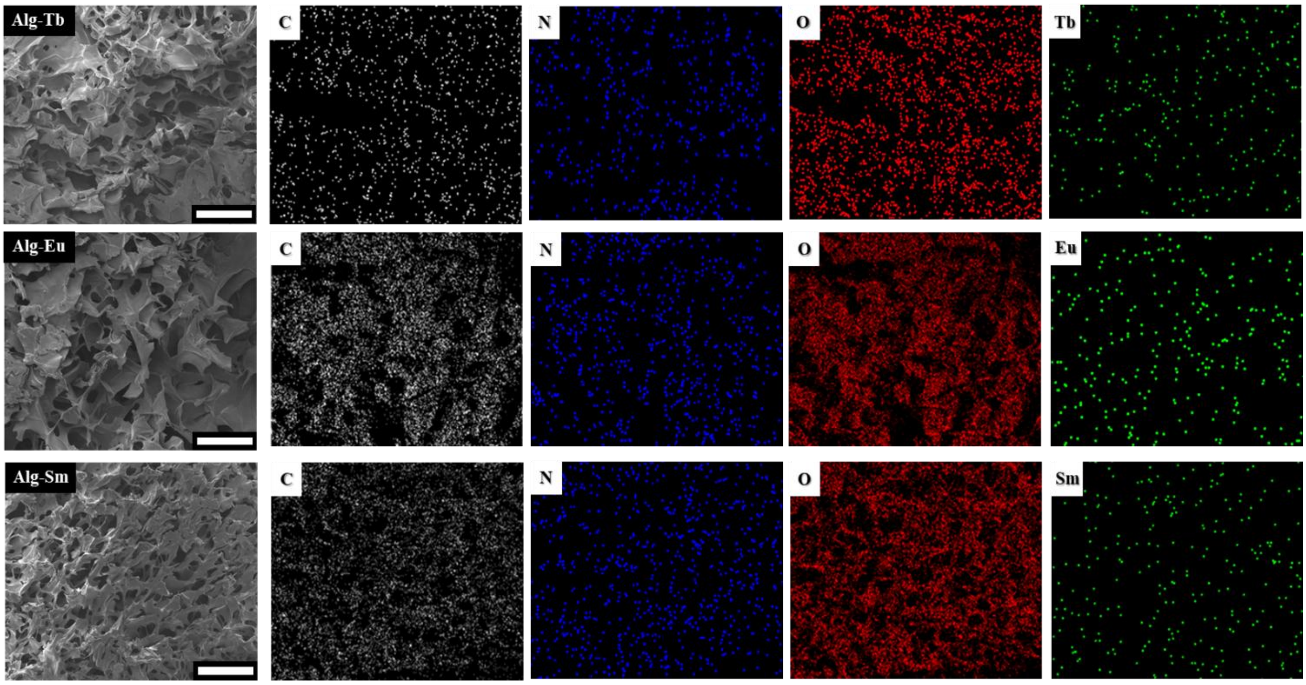

Figure S19. SEM-EDS elemental mapping of Alg-Ln lyophilized hydrogels (Scale bar = 100 μm).

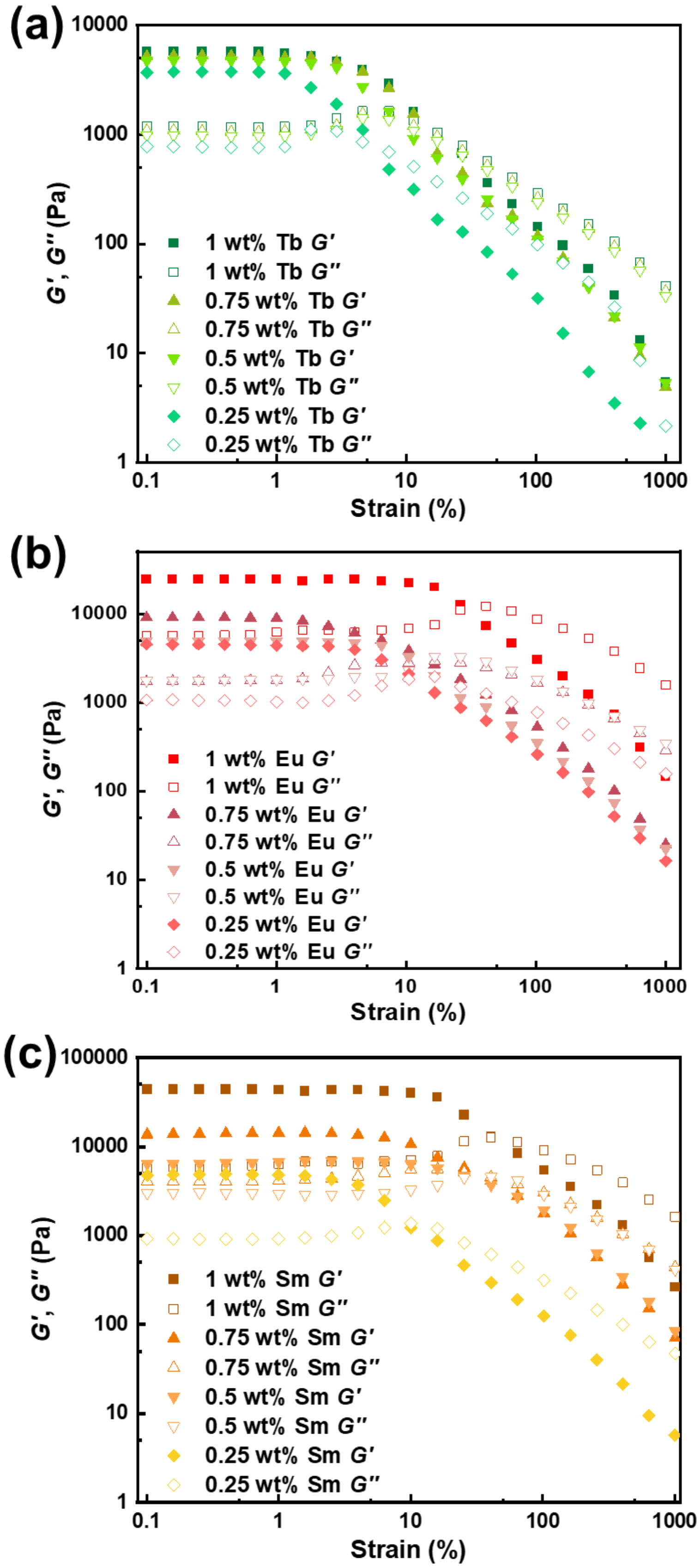

Figure S20. Oscillation strain sweeps of (a) Alg-Tb, (b) Alg-Eu, and (c) Alg-Sm hydrogels.

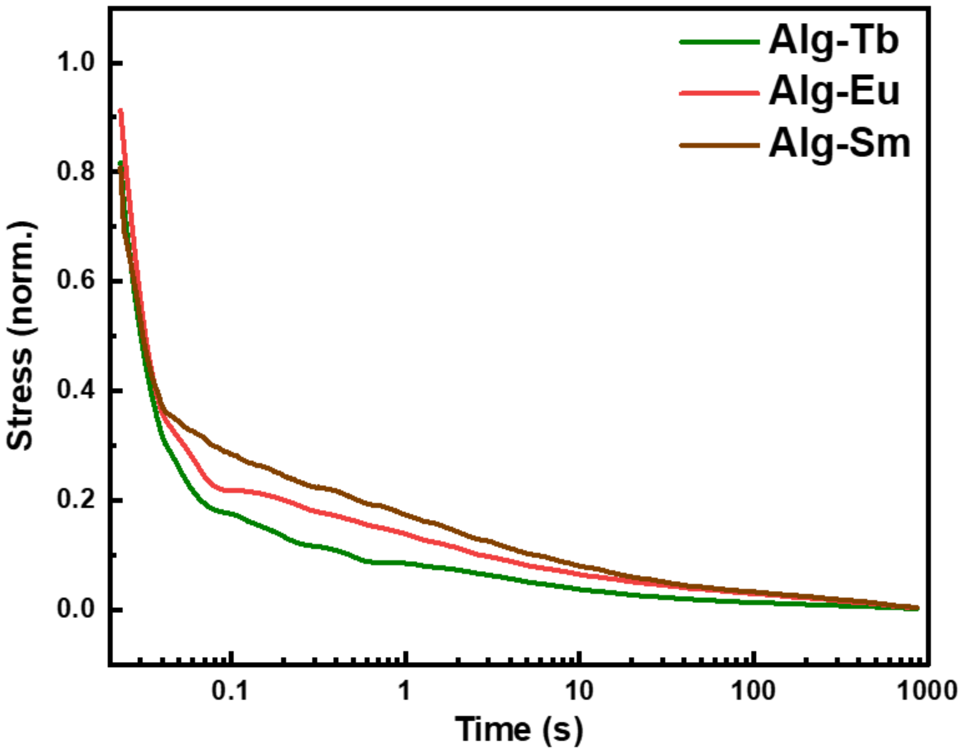

Figure S21. Stress relaxation of Alg-Ln hydrogels.

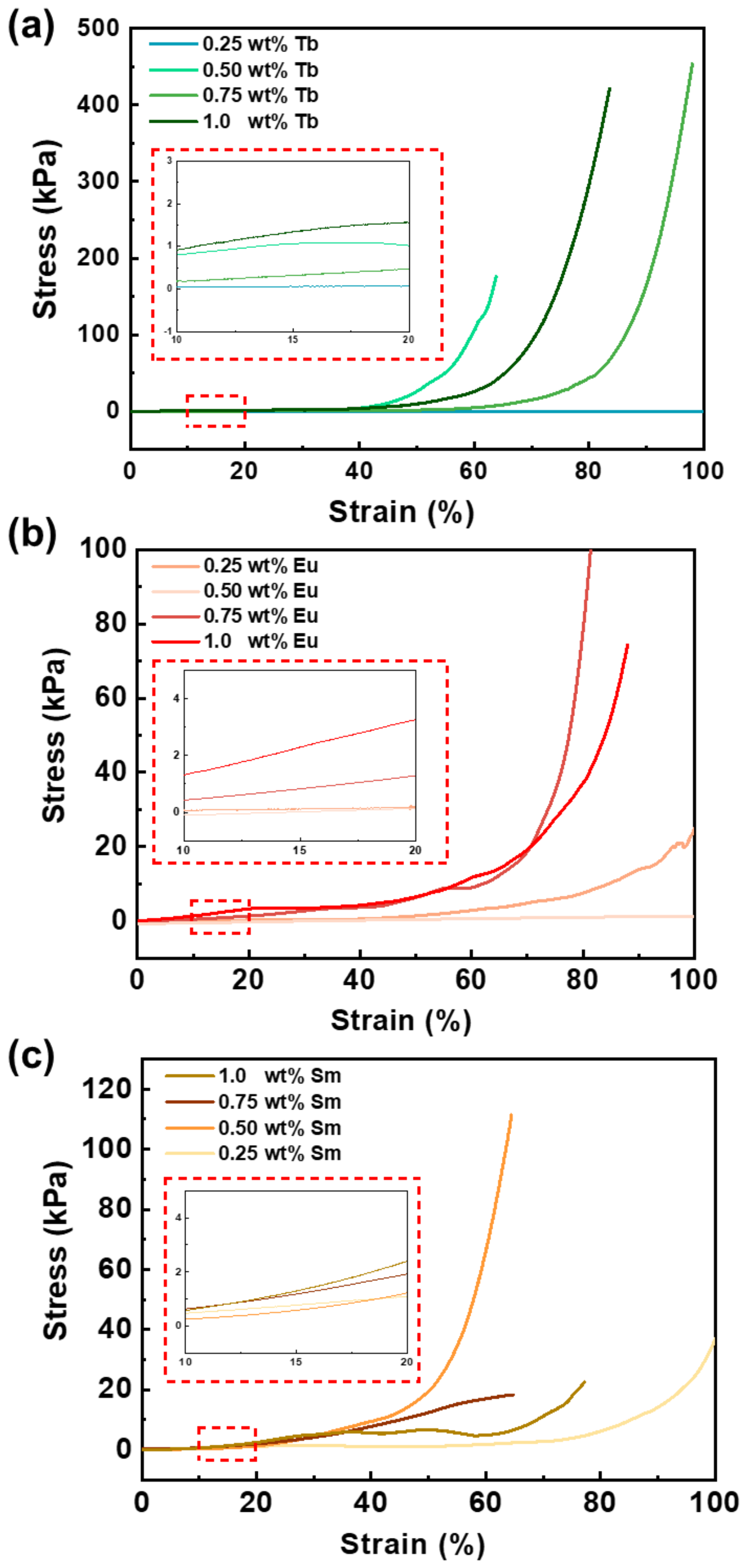

Figure S22. Stress-strain curves of (a) Alg-Tb, (b) Alg-Eu, and (c) Alg-Sm hydrogels.

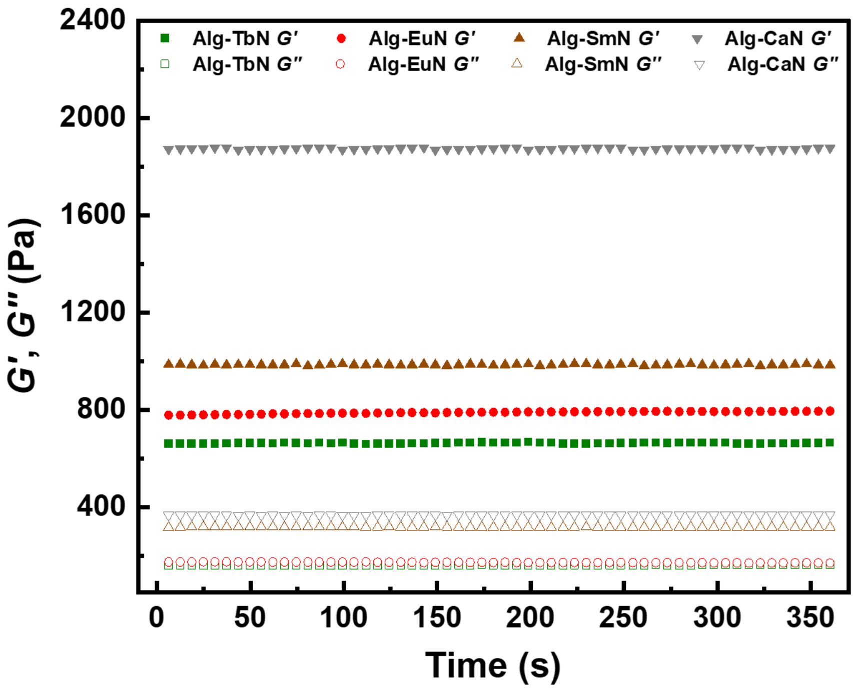

Figure S23. Oscillation time sweeps of hydrogels. Alginate-based hydrogels crosslinked with $Ln(NO_3)_3$ (Alg-LnN) and $CaSO_4$ (Alg-CaN) were prepared using alginate (5 wt%) and crosslinkers (0.5 wt%).

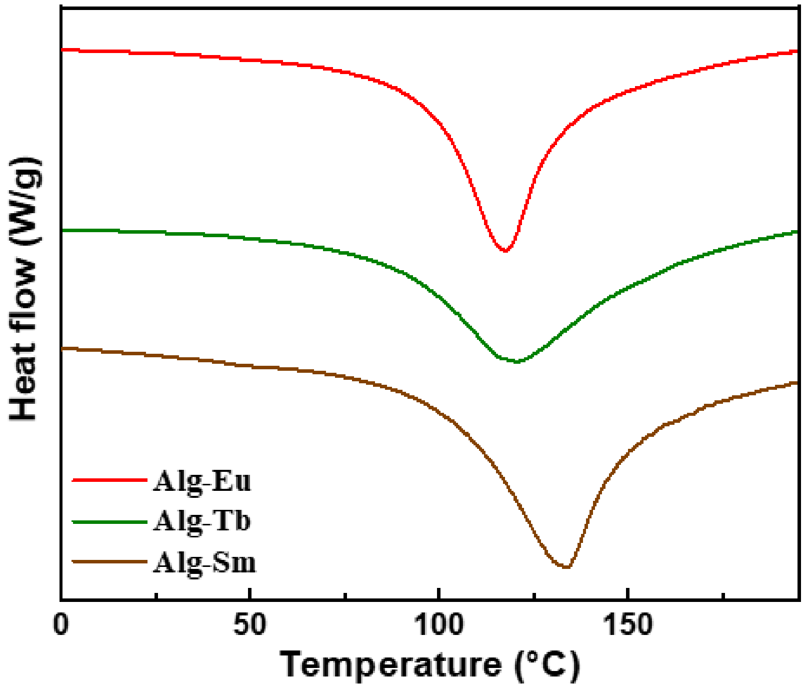

Figure S24. Representative DSC curves of (a) Alg-Tb, (b) Alg-Eu, and (c) Alg-Sm hydrogels.

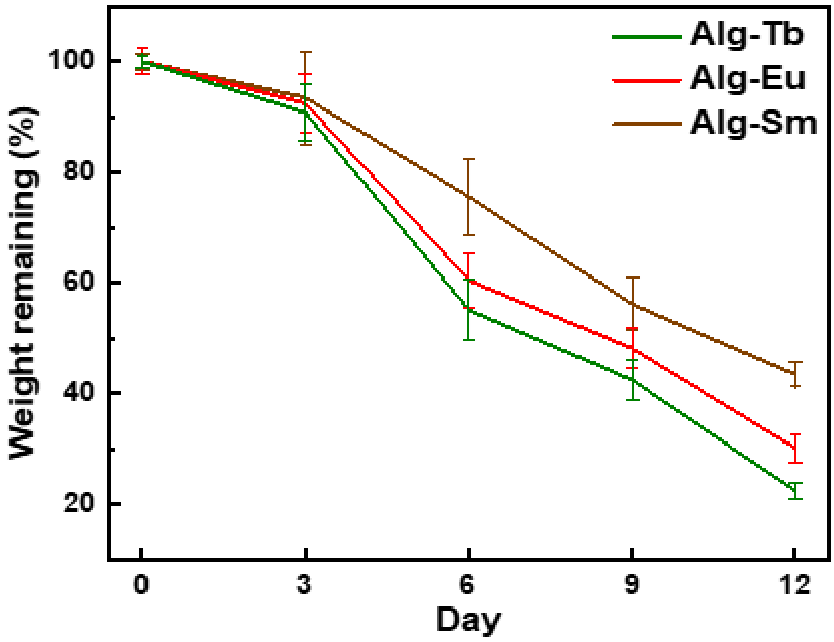

Figure S25. The weight remaining of Alg-Ln hydrogels after immersion.

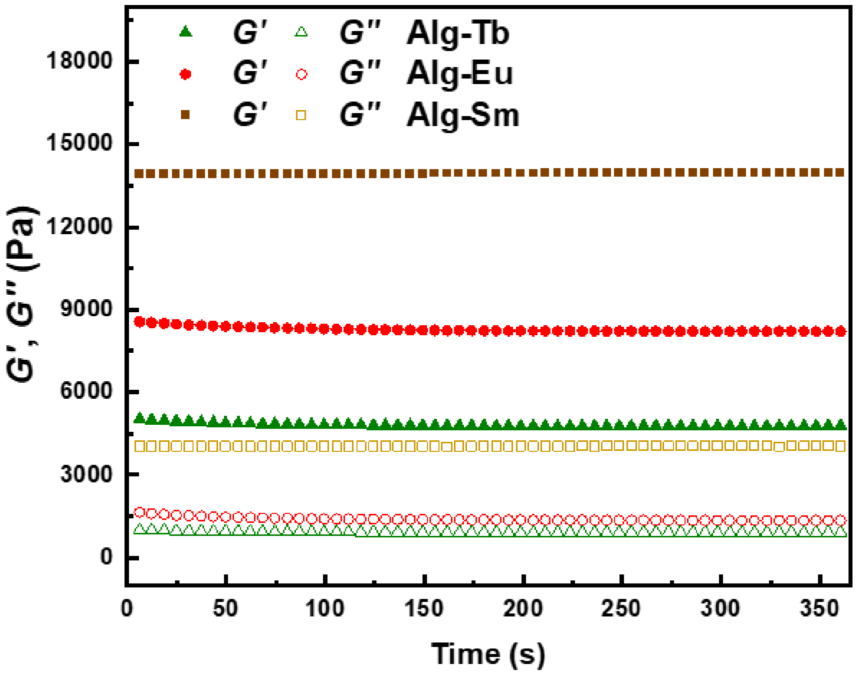

Figure S26. Oscillation-time sweeps of hydrogels after 2 hr of rehydration.

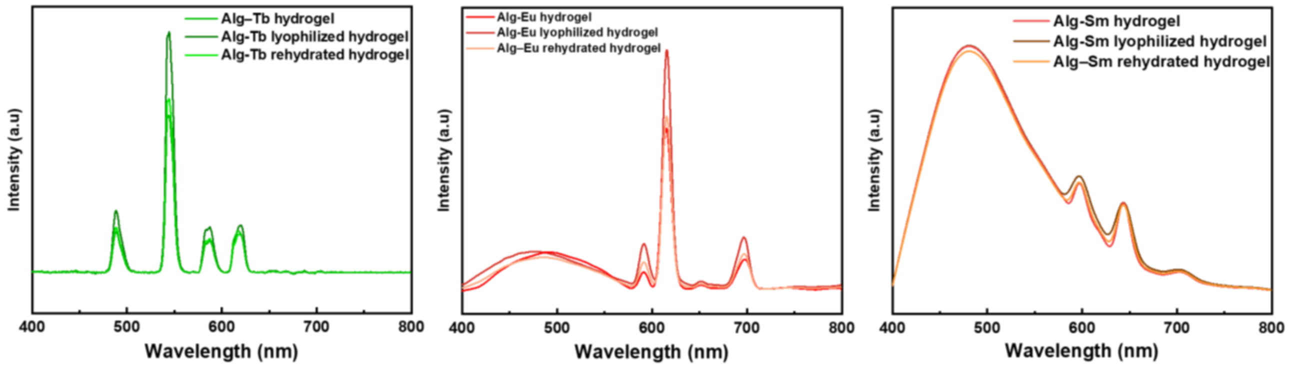

Figure S27. Luminescence spectra of Alg–Ln hydrogels under different states, including lyophilized hydrogel and rehydrated hydrogel (after 2 h immersion in water).

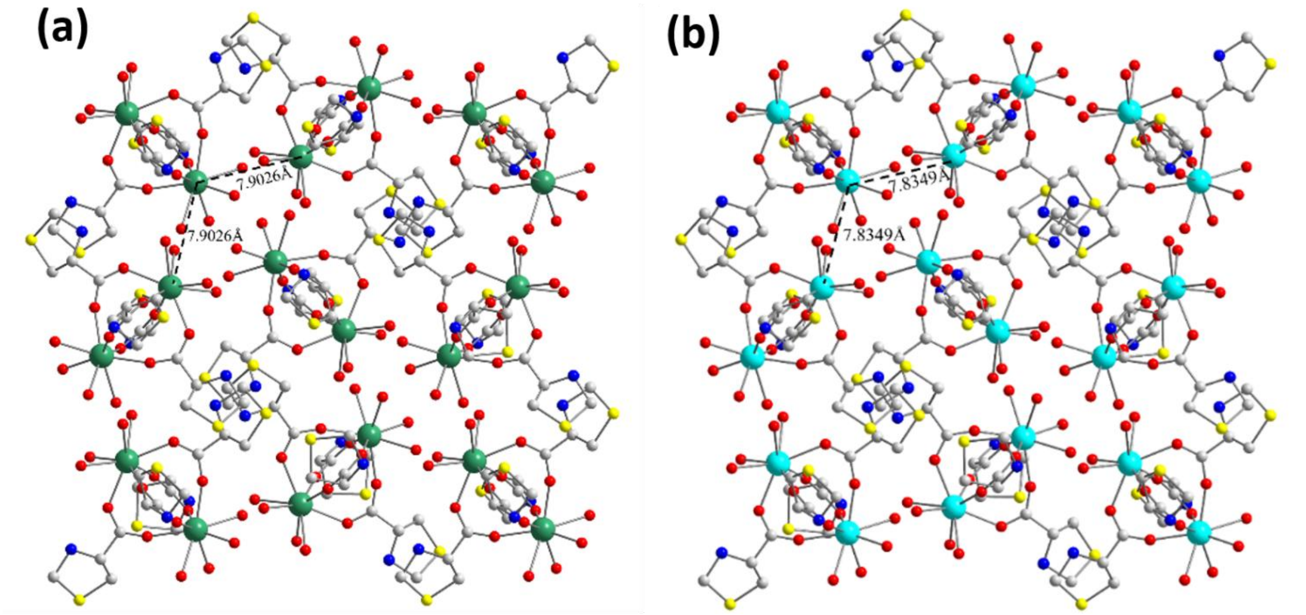

Figure S28. Packing arrangement along the crystallographic *c* axis for (a) **Complex 1** and (b) **Complex 2**. Color code: light blue, Tb; green, Eu; red, O; blue, N; grey, C.; yellow, S. Hydrogen atoms have been omitted for clarity.

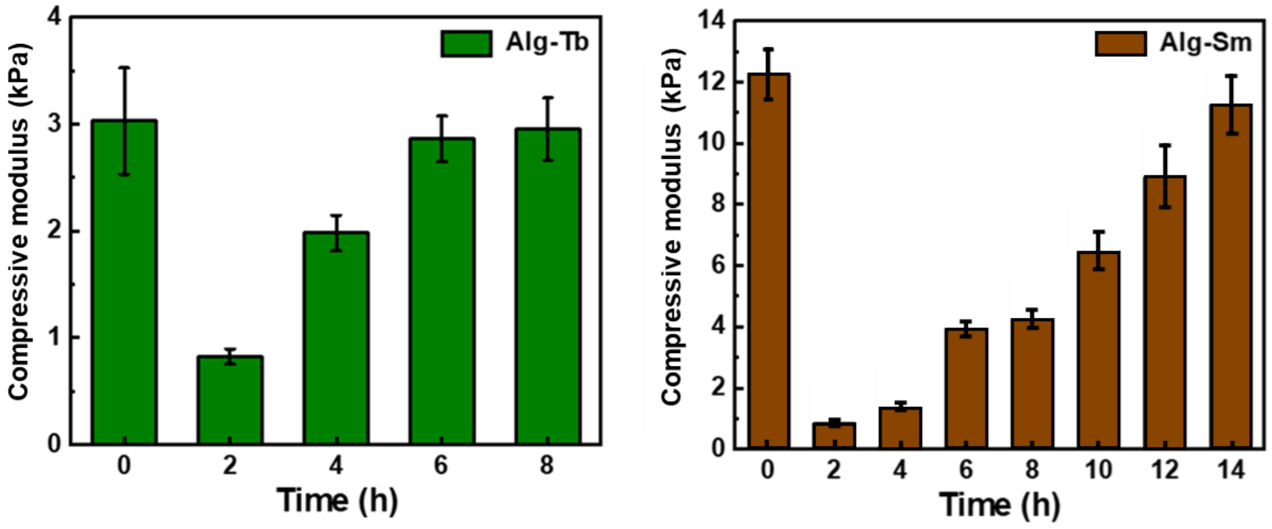

Figure S29. The compressive modulus of hydrogel before and after healing for different time periods.

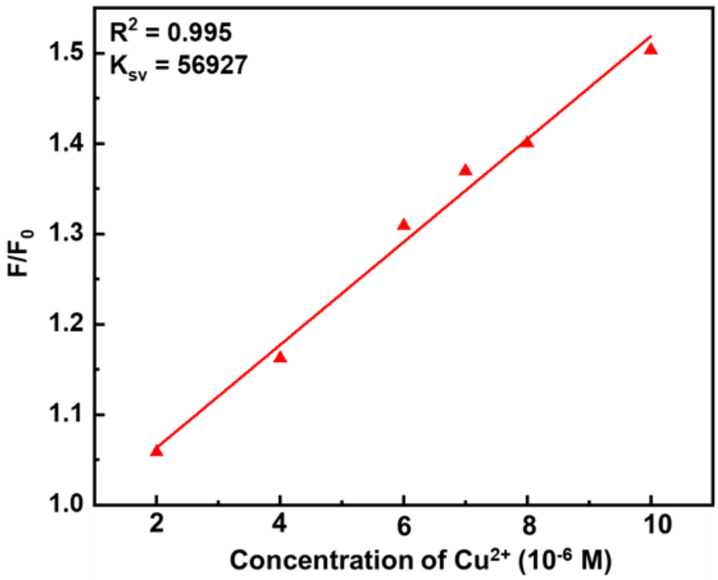

Figure S30. The Stern-Volmer plot of the luminescence quenching of Alg-Eu hydrogels by Cu^{2+} ions.

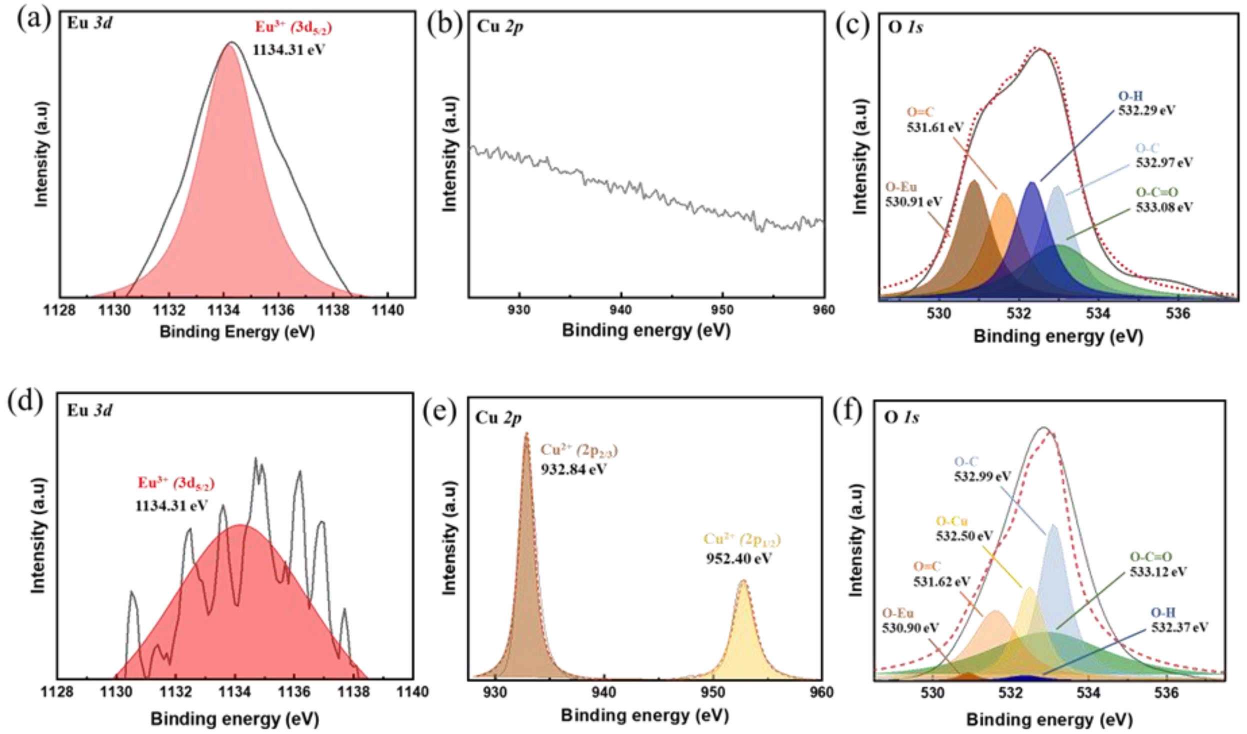

Figure S31. XPS spectra of (a-c) Alg–Eu lyophilized hydrogel and (d-f) Alg–Eu lyophilized hydrogel after Cu^{2+} treatment.

Table S1. Summary of SHAPE1 analysis for complexes **1**

Vertices	Code	Label	Shape	Symmetry
**8**	1	OP-8	Octagon	D_8h
	2	HPY-8	Heptagonal pyramid	C_7v
	3	HBPY-8	Hexagonal bipyramid	D_6h
	4	CU-8	Cube	O_h
	5	SAPR-8	Square antiprism	D_4d
	6	TDD-8	Triangular dodecahedron	D_2d
	7	JGBF-8	Johnson – Gyrobifastigium (J26)	D_2d
	8	JETBPY-8	Johnson – Elongated triangular bipyramid (J14)	D_3h
	9	JBTP-8	Johnson – Biaugmented trigonal prism (J50)	C_2v
	10	BTPR-8	Biaugmented trigonal prism	C_2v
	11	JSD-8	Snub disphenoid (J84)	D_2d
	12	TT-8	Triakis tetrahedron	T_x
	13	ETBPY-8	Elongated trigonal bipyramid (see 8)	D_3h

	OP-8	HPY-8	HBPY-8	CU-8	SAPR-8	TDD-8	JGBF-8	JETBPY-8	JBTPR-8	BTPR-8	JSD-8	TT-8	ETBPY-8
**Tb1**	30.170	24.559	17.161	10.327	2.867	**0.377**	13.337	29.112	2.243	1.636	2.141	10.935	25.201
**Tb2**	27.792	24.661	17.138	11.381	0.838	1.675	14.780	29.268	1.756	1.081	3.750	12.109	24.506

Table S2. Atomic coordinates (x 10^4) and equivalent isotropic displacement parameters ($Å^2$x 10^3) for Tb complex. U(eq) is defined as one third of the trace of the orthogonalized U^{ij} tensor.

	x	y	z	U(eq)
Tb(2)	3127(1)	3127(1)	0	53(1)
Tb(1)	1147(1)	1147(1)	0	58(1)
S(1)	-879(3)	4491(4)	-897(2)	76(1)
S(2)	2834(7)	1253(8)	1905(4)	85(2)
O(5)	1777(7)	3672(8)	-173(5)	58(3)
O(6)	2338(9)	2683(8)	734(5)	66(3)
O(7)	3239(9)	4186(10)	-717(6)	78(4)
O(8)	2986(10)	4445(9)	469(6)	82(4)
O(3)	948(7)	2542(7)	-158(5)	60(3)
O(4)	1362(8)	1693(8)	891(5)	67(3)
O(2)	-196(9)	1559(11)	394(7)	90(5)
O(1)	31(11)	765(13)	-582(8)	112(6)
N(1)	578(9)	4727(10)	-405(5)	57(3)
C(6)	1872(12)	2555(14)	1618(7)	68(4)
C(2)	303(11)	3880(10)	-260(7)	60(4)
C(4)	-213(13)	5237(12)	-525(9)	72(5)
C(7)	2810(30)	2370(50)	1831(17)	78(4)
C(3)	-271(15)	3572(14)	-695(10)	87(7)
C(5)	1863(14)	2257(12)	1032(7)	68(5)
C(1)	1075(11)	3321(13)	-213(6)	62(5)
Cl(2)	4820(3)	1765(3)	1408(1)	57(1)
Cl(1)	857(3)	3300(4)	2794(2)	72(1)
S(2A)	2922(7)	2260(9)	2510(6)	85(2)
Cl(3)	3886(9)	4786(8)	1515(5)	201(6)
C(8)	1710(30)	1150(30)	2171(15)	82(4)
C(8A)	2170(30)	1470(40)	2321(19)	82(4)
C(7A)	2720(30)	2770(60)	1858(19)	78(4)
N(2)	1330(30)	1960(30)	1932(18)	71(5)
N(2A)	1510(40)	1970(50)	2030(30)	71(5)

Table S3. Bond lengths [Å] and angles [°] for Tb complex.

Tb(2)-O(5)	2.364(12)	C(8A)-N(2A)	1.50(3)
Tb(2)-O(5)#1	2.364(12)		
Tb(2)-O(6)#1	2.314(12)	O(5)-Tb(2)-O(5)#1	134.8(6)
Tb(2)-O(6)	2.314(12)	O(5)-Tb(2)-O(7)	71.5(4)
Tb(2)-O(7)#1	2.449(13)	O(5)#1-Tb(2)-O(7)	137.1(4)
Tb(2)-O(7)	2.449(13)	O(5)#1-Tb(2)-O(7)#1	71.5(4)
Tb(2)-O(8)#1	2.411(14)	O(5)-Tb(2)-O(7)#1	137.1(4)
Tb(2)-O(8)	2.411(14)	O(5)-Tb(2)-O(8)	71.3(5)
Tb(1)-O(3)#1	2.282(11)	O(5)-Tb(2)-O(8)#1	138.1(5)
Tb(1)-O(3)	2.282(11)	O(5)#1-Tb(2)-O(8)	138.1(5)
Tb(1)-O(4)	2.385(12)	O(5)#1-Tb(2)-O(8)#1	71.3(5)
Tb(1)-O(4)#1	2.385(12)	O(6)-Tb(2)-O(5)	75.9(4)
Tb(1)-O(2)#1	2.443(14)	O(6)-Tb(2)-O(5)#1	77.3(5)
Tb(1)-O(2)	2.443(14)	O(6)#1-Tb(2)-O(5)	77.3(5)
Tb(1)-O(1)	2.367(16)	O(6)#1-Tb(2)-O(5)#1	75.9(4)
Tb(1)-O(1)#1	2.367(16)	O(6)#1-Tb(2)-O(6)	106.0(6)
S(1)-C(4)	1.84(2)	O(6)#1-Tb(2)-O(7)	80.5(5)
S(1)-C(3)	1.83(2)	O(6)-Tb(2)-O(7)	144.4(5)
S(2)-C(7)	1.79(8)	O(6)#1-Tb(2)-O(7)#1	144.4(5)
S(2)-C(8)	1.92(4)	O(6)-Tb(2)-O(7)#1	80.5(5)
O(5)-C(1)	1.26(2)	O(6)#1-Tb(2)-O(8)	145.1(5)
O(6)-C(5)	1.26(2)	O(6)#1-Tb(2)-O(8)#1	81.0(5)
O(3)-C(1)	1.27(2)	O(6)-Tb(2)-O(8)#1	145.1(5)
O(4)-C(5)	1.25(3)	O(6)-Tb(2)-O(8)	81.0(5)
N(1)-C(2)	1.47(2)	O(7)#1-Tb(2)-O(7)	114.7(7)
N(1)-C(4)	1.53(2)	O(8)-Tb(2)-O(7)	75.5(5)
C(6)-C(7)	1.61(5)	O(8)-Tb(2)-O(7)#1	69.9(5)
C(6)-C(5)	1.52(2)	O(8)#1-Tb(2)-O(7)	69.9(5)
C(6)-C(7A)	1.52(6)	O(8)#1-Tb(2)-O(7)#1	75.5(5)
C(6)-N(2)	1.50(4)	O(8)-Tb(2)-O(8)#1	113.1(8)
C(6)-N(2A)	1.50(3)	O(3)-Tb(1)-O(3)#1	107.5(6)
C(2)-C(3)	1.49(3)	O(3)#1-Tb(1)-O(4)#1	79.6(5)
C(2)-C(1)	1.525(19)	O(3)-Tb(1)-O(4)	79.6(5)
S(2A)-C(8A)	1.79(7)	O(3)#1-Tb(1)-O(4)	75.8(4)
S(2A)-C(7A)	1.83(6)	O(3)-Tb(1)-O(4)#1	75.8(4)
C(8)-N(2)	1.54(7)	O(3)#1-Tb(1)-O(2)	145.7(5)

O(3)#1-Tb(1)-O(2)#1	71.5(5)	C(5)-O(4)-Tb(1)	127.5(11)
O(3)-Tb(1)-O(2)#1	145.7(5)	C(2)-N(1)-C(4)	106.9(14)
O(3)-Tb(1)-O(2)	71.5(5)	C(5)-C(6)-C(7)	105(2)
O(3)-Tb(1)-O(1)	92.5(6)	C(5)-C(6)-C(7A)	117(3)
O(3)#1-Tb(1)-O(1)#1	92.5(6)	N(2)-C(6)-C(7)	105(3)
O(3)#1-Tb(1)-O(1)	143.3(5)	N(2)-C(6)-C(5)	107(2)
O(3)-Tb(1)-O(1)#1	143.3(5)	N(2A)-C(6)-C(5)	117(4)
O(4)-Tb(1)-O(4)#1	137.8(6)	N(2A)-C(6)-C(7A)	103(4)
O(4)-Tb(1)-O(2)#1	130.3(5)	N(1)-C(2)-C(3)	108.3(15)
O(4)#1-Tb(1)-O(2)#1	70.3(5)	N(1)-C(2)-C(1)	108.4(15)
O(4)-Tb(1)-O(2)	70.3(5)	C(3)-C(2)-C(1)	111.0(15)
O(4)#1-Tb(1)-O(2)	130.3(5)	N(1)-C(4)-S(1)	103.2(12)
O(2)-Tb(1)-O(2)#1	129.0(8)	C(6)-C(7)-S(2)	104(3)
O(1)#1-Tb(1)-O(4)#1	139.4(5)	C(2)-C(3)-S(1)	104.9(13)
O(1)-Tb(1)-O(4)	139.4(5)	O(6)-C(5)-C(6)	112.2(19)
O(1)-Tb(1)-O(4)#1	75.8(6)	O(4)-C(5)-O(6)	127.8(16)
O(1)#1-Tb(1)-O(4)	75.8(6)	O(4)-C(5)-C(6)	119.6(18)
O(1)-Tb(1)-O(2)	69.4(6)	O(5)-C(1)-O(3)	124.9(15)
O(1)-Tb(1)-O(2)#1	74.7(6)	O(5)-C(1)-C(2)	117.8(18)
O(1)#1-Tb(1)-O(2)	74.7(6)	O(3)-C(1)-C(2)	116.9(17)
O(1)#1-Tb(1)-O(2)#1	69.4(6)	C(8A)-S(2A)-C(7A)	88(3)
O(1)#1-Tb(1)-O(1)	88.8(11)	N(2)-C(8)-S(2)	100(3)
C(3)-S(1)-C(4)	94.4(9)	N(2A)-C(8A)-S(2A)	103(5)
C(7)-S(2)-C(8)	96(2)	C(6)-C(7A)-S(2A)	113(4)
C(1)-O(5)-Tb(2)	131.5(12)	C(6)-N(2)-C(8)	120(4)
C(5)-O(6)-Tb(2)	162.0(12)	C(6)-N(2A)-C(8A)	112(4)
C(1)-O(3)-Tb(1)	162.4(11)		

Symmetry transformations used to generate equivalent atoms:

#1 y,x,-z

Table S4. Anisotropic displacement parameters (Å^{2}x 10^3) for Tb complex.The anisotropic displacement factor exponent takes the form: $-2p^2[h^2 a^{*2}U^{11} + ... + 2 h k a^* b^* U^{12}]$

	U^{11}	U^{22}	U^{33}	U^{23}	U^{13}	U^{12}
Tb(2)	52(1)	52(1)	55(1)	10(1)	-10(1)	-6(1)
Tb(1)	48(1)	48(1)	79(1)	-8(1)	8(1)	-7(1)
S(1)	69(3)	92(4)	69(3)	6(2)	-11(2)	8(3)
S(2)	73(4)	122(6)	62(3)	11(4)	0(3)	28(4)
O(5)	53(6)	68(7)	53(6)	-6(5)	2(5)	-9(6)
O(6)	88(9)	66(7)	45(6)	4(5)	-2(6)	-9(7)
O(7)	61(8)	83(9)	89(9)	23(8)	-8(7)	12(7)
O(8)	95(11)	56(7)	95(10)	-3(7)	-30(8)	-10(7)
O(3)	54(6)	48(6)	79(8)	0(5)	7(5)	11(5)
O(4)	81(8)	58(7)	62(7)	-16(6)	15(6)	-6(6)
O(2)	64(9)	97(12)	108(12)	-10(10)	27(9)	-14(8)
O(1)	72(9)	127(13)	136(14)	-61(11)	2(9)	-14(9)
N(1)	54(8)	75(9)	42(6)	-7(6)	-5(6)	15(6)
C(6)	54(7)	105(10)	45(7)	4(7)	-1(6)	20(8)
C(2)	73(11)	56(9)	52(8)	8(7)	-6(8)	-10(8)
C(4)	74(12)	62(11)	80(13)	-9(9)	8(10)	21(9)
C(7)	65(8)	116(9)	52(7)	13(8)	3(6)	25(8)
C(3)	84(15)	82(14)	96(15)	-31(12)	-35(12)	11(11)
C(5)	90(13)	64(11)	49(8)	5(8)	-2(9)	36(10)
C(1)	62(11)	98(15)	25(6)	-2(8)	0(6)	16(10)
Cl(2)	59(2)	68(2)	43(2)	-3(2)	-11(2)	3(2)
Cl(1)	64(3)	101(4)	52(2)	-1(2)	-2(2)	3(2)
S(2A)	73(4)	122(6)	62(3)	11(4)	0(3)	28(4)
Cl(3)	237(12)	169(9)	197(10)	-53(8)	-129(9)	93(9)
C(8)	72(8)	113(9)	60(8)	10(8)	3(7)	31(8)
C(8A)	72(8)	113(9)	60(8)	10(8)	3(7)	31(8)
C(7A)	65(8)	116(9)	52(7)	13(8)	3(6)	25(8)
N(2)	61(10)	103(9)	50(9)	8(8)	6(8)	26(9)
N(2A)	61(10)	103(9)	50(9)	8(8)	6(8)	26(9)

Table S5. Hydrogen coordinates (x 10^4) and isotropic displacement parameters ($Å^2$x 10^3) for Tb complex.

	x	y	z	U(eq)
H(7A)	3272	4702	-573	116
H(7B)	2791	4170	-937	116
H(8A)	2967	4904	233	123
H(8B)	3432	4548	706	123
H(2A)	-483	1291	365	135
H(2B)	-176	2085	551	135
H(1A)	-480	785	-409	168
H(1B)	92	229	-715	168
H(1C)	916	4712	-703	68
H(1D)	868	4962	-125	68
H(2)	0	3894	95	72
H(4A)	-83	5733	-750	86
H(4B)	-487	5423	-185	86
H(7C)	2912	2652	2182	94
H(7D)	3230	2562	1563	94
H(3A)	-642	3125	-555	105
H(3B)	52	3349	-1007	105
H(8C)	1429	644	2029	98
H(8D)	1690	1149	2573	98
H(8AA)	2428	1054	2075	98
H(8AB)	1946	1186	2644	98
H(7AA)	2758	3389	1908	94
H(7AB)	3167	2609	1597	94
H(2C)	899	1804	1710	86
H(2D)	1099	2251	2212	86
H(2AA)	1145	1616	1870	86
H(2AB)	1218	2277	2284	86

Table S6. Torsion angles [°] for Tb complex.

Tb(2)-O(5)-C(1)-O(3)	1(2)	C(3)-C(2)-C(1)-O(5)	134.9(18)
Tb(2)-O(5)-C(1)-C(2)	172.8(10)	C(3)-C(2)-C(1)-O(3)	-52(2)
Tb(2)-O(6)-C(5)-O(4)	-18(5)	C(5)-C(6)-C(7)-S(2)	-73(2)
Tb(2)-O(6)-C(5)-C(6)	168(3)	C(5)-C(6)-C(7A)-S(2A)	-129(3)
Tb(1)-O(3)-C(1)-O(5)	3(5)	C(5)-C(6)-N(2)-C(8)	87(4)
Tb(1)-O(3)-C(1)-C(2)	-169(3)	C(5)-C(6)-N(2A)-C(8A)	98(7)
Tb(1)-O(4)-C(5)-O(6)	3(3)	C(1)-C(2)-C(3)-S(1)	-156.4(14)
Tb(1)-O(4)-C(5)-C(6)	176.5(11)	S(2A)-C(8A)-N(2A)-C(6)	49(7)
S(2)-C(8)-N(2)-C(6)	-3(4)	C(8)-S(2)-C(7)-C(6)	-38(3)
N(1)-C(2)-C(3)-S(1)	-38(2)	C(8A)-S(2A)-C(7A)-C(6)	23(5)
N(1)-C(2)-C(1)-O(5)	16(2)	C(7A)-C(6)-C(5)-O(6)	-43(4)
N(1)-C(2)-C(1)-O(3)	-171.3(13)	C(7A)-C(6)-C(5)-O(4)	143(4)
C(2)-N(1)-C(4)-S(1)	-39.8(15)	C(7A)-C(6)-N(2A)-C(8A)	-31(8)
C(4)-S(1)-C(3)-C(2)	11.4(18)	C(7A)-S(2A)-C(8A)-N(2A)	-38(4)
C(4)-N(1)-C(2)-C(3)	51.9(19)	N(2)-C(6)-C(7)-S(2)	39(3)
C(4)-N(1)-C(2)-C(1)	172.4(13)	N(2)-C(6)-C(5)-O(6)	-176(3)
C(7)-C(6)-C(5)-O(6)	-66(3)	N(2)-C(6)-C(5)-O(4)	10(3)
C(7)-C(6)-C(5)-O(4)	120(3)	N(2A)-C(6)-C(5)-O(6)	-165(3)
C(7)-C(6)-N(2)-C(8)	-24(5)	N(2A)-C(6)-C(5)-O(4)	21(3)
C(3)-S(1)-C(4)-N(1)	15.7(15)	N(2A)-C(6)-C(7A)-S(2A)	0(6)

Symmetry transformations used to generate equivalent atoms:

#1 y,x,-z

Table S7. The PLQY values of Alg-Ln samples.

		PLQY (%)
**Alg-Tb**	Wet hydrogel	0.17 ± 0.06
	Lyophilized hydrogel	0.20 ± 0.03
	Rehydrated hydrogel (immersion in water for 2 hrs)	0.18 ± 0.05
**Alg-Eu**	Wet hydrogel	2.01± 0.07
	Lyophilized hydrogel	2.51 ± 0.06
	Rehydrated hydrogel (immersion in water for 2 hrs)	2.17 ± 0.07
**Alg-Sm**	Wet hydrogel	0.06 ± 0.01
	Lyophilized hydrogel	0.07 ± 0.02
	Rehydrated hydrogel (immersion in water for 2 hrs)	0.06 ± 0.02

Table S8. The PLQY values of Alg-LnN samples.

		PLQY (%)
**Alg-TbN**	Wet hydrogel	0.18 ± 0.01
	Lyophilized hydrogel	0.21 ± 0.01
**Alg-EuN**	Wet hydrogel	1.01 ± 0.08
	Lyophilized hydrogel	1.22 ± 0.09
**Alg-SmN**	Wet hydrogel	0.08± 0.01
	Lyophilized hydrogel	0.12 ± 0.01

Table S9. Micro-CT analyses of Alg-Ln hydrogels with 0.75 wt% concentration of Ln complex.

	**Pore size (μm)**	**Closed porosity (%)**	**Open porosity (%)**	**Total porosity (%)**
**Alg-Tb**	116	0.077	78.7	78.9
**Alg-Eu**	93	0.056	81.8	81.9
**Alg-Sm**	79	0.097	78.9	78.9

Table S10. MIP analyses of Alg-Ln hydrogels with 0.75 wt% concentration of Ln complex.

	**Total intrusion volume (mL/g)**	**Total pore area (m^2/g)**	**Median pore diameter (μm)**	**Porosity (%)**
**Alg-Tb**	10.81	1.07	52.16	98.21
**Alg-Eu**	9.89	1.03	46.35	98.81
**Alg-Sm**	8.77	0.89	44.95	98.15

Table S11. Equilibrium rheological properties of Alg–Ln hydrogels (with 0.25–1.0 wt% Ln complexes)

	**Concentration of Ln complex (wt%)**	***G'* (Pa)**	***G''* (Pa)**	**Crosslinking density ($mol \cdot m^{-3}$)**	**Flow point (%)**
**Alg-Tb**	0.25	3706 ± 210	7765 ± 439	1.50 ± 0.08	5.55 ± 0.19
	0.50	4645 ± 396	977 ± 84	1.87 ± 0.19	8.98 ± 0.24
	0.75	5212 ± 444	1062 ± 91	2.04 ± 0.18	13.75 ± 0.73
	1.0	5708 ± 361	1199 ± 77	2.30 ± 0.14	15.79 ± 0. 56
**Alg-Eu**	0.25	4508 ± 389	1061 ± 92	1.82 ± 0.16	11.66 ± 0.63
	0.50	4893 ± 207	1802 ± 82	1.97 ± 0.08	12.65 ± 0.71
	0.75	8820 ± 664	1763 ± 134	3.56 ± 0.27	15.28 ± 0.20
	1.0	24465 ± 2084	5594 ± 483	9.87 ± 0.84	28.51 ± 1.29
**Alg-Sm**	0.25	4829 ± 222	920 ± 46	1.95 ± 0.10	9.41 ± 0.43
	0.50	6606 ± 362	3005 ± 161	2.67 ± 0.15	28.52 ± 1.36
	0.75	14283 ± 1218	4140 ± 346	5.76 ± 0.49	32.46 ± 2.14
	1.0	43623 ± 3601	6402 ± 529	17.61 ± 1.45	41.52 ± 2.34

Table S12. Rheological properties of Alg–Ln hydrogels before and after rehydration in water for 2 hr.

		***G'* (Pa)**	***G''* (Pa)**
**Alg-Tb**	Wet hydrogel	5212 ± 444	1062 ± 91
	Rehydrated hydrogel	4811 ± 360	932 ± 81
**Alg-Eu**	Wet hydrogel	8820 ± 664	1763 ± 134
	Rehydrated hydrogel	8282 ± 452	1399 ± 125
**Alg-Sm**	Wet hydrogel	14283 ± 1218	4140 ± 346
	Rehydrated hydrogel	13948 ± 1101	4047 ± 349
